# Synthesis and antimicrobial evaluation of a new hybrid bis-cyanoacrylamide-based-piperazine containing sulphamethoxazole moiety against rheumatoid arthritis-associated pathogens

**DOI:** 10.1007/s00210-024-03780-7

**Published:** 2025-01-20

**Authors:** Mona M. Soliman, Ahmed H. M. Elwahy, Ahmed M. Sayed, Mahmoud Ibrahim, Mohamed A. Dawoud, Shahd Hisham Mohamed Ali, Menna Tallah S. Nady, Nada A. Hassan, Wessam Saad, Ismail A. Abdelhamid

**Affiliations:** 1 Department of Botany and Microbiology, Faculty of Science, Cairo University, Giza, 12613 Egypt; 2 Department of Chemistry, Faculty of Science, Cairo University, Giza, 12613 Egypt; 3 Department of Biotechnology, Faculty of Agriculture, Cairo University, Giza, 12613 Egypt; 4 Department of Zoology, Faculty of Science, Cairo University, Giza, 12613 Egypt

**Keywords:** Bis-cyanoacrylamide-based-piperazine, Sulphamethoxazole, Rheumatoid arthritis, Antibacterial agent, Antifungal agent, Molecular docking

## Abstract

Piperazine-based compounds have garnered significant attention due to their notable biological and pharmacological activities, making them essential in fine chemical and pharmaceutical applications. In this study, we managed to synthesize a novel hybrid bis-cyanoacrylamide bearing the piperazine core via phenoxymethyl linker and incorporating sulphamethoxazole moiety. The novel compound was fully characterized using different spectral data including 1H-NMR, ^13^C-NMR, and FTIR spectroscopy. Piperazine-based compounds were screened for in silico studies to understand the antimicrobial activity against infections that may contribute to rheumatoid arthritis symptoms. The tested piperazine compound was also evaluated for its antimicrobial activity against *Aspergillus niger*, *Candida albicans*, *Enterococcus faecalis* ATCC 29212, *Staphylococcus aureus* ATCC 29213, *Pseudomonas aeuroginosa* ATCC 27853, *Escherichia coli* ATCC 25922, and *Klebsiella pneumoniae* ATCC 700603. *S. aureus* showed the highest inhibition, with a zone diameter of 16.0 ± 1.0 mm at a concentration of 0.8 mg/ml. The minimal inhibitory concentration (MIC) for all bacterial species ranged from 5 to 40 mg/ml. In contrast, fungal species were the most resistant to the tested compound. Molecular docking studies were conducted to elucidate the interaction mechanisms, binding energies, and hydrogen bonding interactions within protein–ligand complexes. Molecular docking studies were performed against five bacterial proteins and two fungal proteins, including DNA gyrase subunit B (UniProt ID: Q839Z1), protein RecA of (UniProt ID: P0A7G6), cyclic AMP-AMP-AMP synthase (UniProt ID: P0DTF7), UDP-N-acetylglucosamine 1-carboxyvinyl transferase (UniProt ID: A0A1S5RKE3), and clumping factor A (UniProt ID: Q53653). The tested compound achieved the highest binding score of ∆G =  − 10.9 kcal/mol at the cyclic AMP synthase active site (UniProt ID: P0DTF7), forming 26 interactions. The results demonstrated that the synthesized piperazine compound exhibits promising antibacterial and antifungal activities, highlighting its potential as a candidate for antimicrobial development.

**Supplementary Information:**

The online version contains supplementary material available at 10.1007/s00210-024-03780-7.

## Introduction

Rheumatoid arthritis (RA) is a chronic inflammatory autoimmune disease that affects symmetric joints. The disease is characterized by persistent joint pain, tenderness, swelling, rigidity, and stiffness, with bone and cartilage damage (Padyukov 2022; Sokolova et al. 2022). People with a history of RA infection may have a higher risk of mortality with a percentage of 52% than those without such a history. The gut microbiome, composed of bacterial and fungal species, plays a significant role in RA development. Differences in gut microbiota composition have been observed between RA patients and healthy individuals. Therefore, in RA patients, the beneficial bacteria decrease while the pathogenic bacteria increase, which can worsen the disease (Dong et al. 2023). The shift in the gut microbiota composition appears to be closely linked to the development of RA, as pathogenic bacteria, either alone or through interactions with other bacteria, may play a direct role in the disease’s progression (Afrasiabi et al. 2023). For instance, certain fungal species like *Saccharomyces cerevisiae* and *Candida albicans* might contribute to the progression of RA (Dagar et al. 2023). However, *Aspergillus* species are not directly associated with joint invasion in RA (Abdel-Azeem et al. 2016). Furthermore, several bacterial species, including *Klebsiella, Escherichia*, *Eisenbergiella*, *Shigella*, *Flavobacterium*, *Enterobacter*, *Staphylococcus aureus*, *Neisseria gonorrhea*, and *Pseudomonas aeruginosa*, were found to be more prevalent in RA patients compared to healthy individuals. On the other hand, *Enterococcus* was more common in healthy controls (Radu and Bungau 2021; Yu et al. 2022).

In treating RA, various pharmaceutical and antimicrobial agents, including methotrexate (MTX), corticosteroids (GCs), nonsteroidal anti-inflammatory drugs (NSAIDs), and disease-modifying anti-rheumatic drugs (DMARDs), are used to suppress the immune system and decrease inflammation (Radu and Bungau 2021). Aggressive RA therapy raises the risk of respiratory infections even while it improves disease outcomes and quality of life (Tsuchiya and Sagara 2018). Additionally, antibiotics like sulfasalazine (SASP) and tetracycline, which have a broad spectrum of antibiotic action, are often used to treat infections that may contribute to RA symptoms. Ciprofloxacin and itraconazole were drugs for treating different bacterial and fungal infections and had therapeutic effects on humans and animals in clinical therapy (Feng et al. 2021). Nevertheless, they can cause severe side effects including urticaria, photosensitivity, neutropenia, lymphopenia, thrombocytopenia, hepatotoxicity, vomiting, diarrhea, anorexia, and the suppression of spermatogenesis (Ogrendik 2013).

Therefore, there is a pressing need for innovative treatments to target RA-associated bacteria and provide safer alternatives (Afrasiabi et al. 2023).

Moreover, piperazine scaffolds, a category of heterocyclic molecules, have applications in medicinal chemistry because of their adaptable core structure (Al-Ghorbani et al. 2015; Shaquiquzzaman et al. 2015; Zhang et al. 2021). Their biological uses include antibacterial, antifungal, antiviral, antidiabetic, antioxidant, antimalarial, and anticancer activities (Lamb 2015; He et al. 2017; Cao et al. 2018; Ji et al. 2019; Sergeant et al. 2019). Besides, it was found that the incorporation of nitrogen atoms in piperazine improves pharmacokinetic properties and water solubility (Lacivita et al. 2009). This makes it a valuable starting point for developing new medications.

Besides, acrylamide-containing compounds have pronounced biological uses as anticancer (Fadda et al. 2012), antidiabetic (Maren 1976), antimicrobial, (El-Gaby et al. 2000), anti-inflammatory (Roifman et al. 2000), and antifungal properties (El-Gaby et al. 2002). Specifically, 3-aryl-2-cyanoacrylamide scaffolds with sulfonamide moieties are used as inhibitors of the metalloenzyme carbonic anhydrase and against the trans-membrane, tumor-associated CA IX and XII and cytosolic human (h) isoforms hCA I and II, which are confirmed antitumor targets (Alafeefy et al. 2013).

Furthermore, significant interest in sulphamethoxazole moiety has been encouraged by several potential pharmacological activities, such as anticancer (Ghorab et al. 2009; Gupta et al. 2013) and antimicrobial agents (Zhanel et al. 2000; Hida et al. 2005; Zander et al. 2010; Underwood et al. 2011; Eldesouky et al. 2018; Tondolo et al. 2018; Hassan et al. 2022, 2023, 2024a, c, b; Abdullah et al. 2024).

Thus, it is believed that integrating the aforementioned cyanoacrylamide, piperazine, and sulphamethoxazole in a basic context may yield molecules with significant biological properties.

Depending on the preceding assumptions, and in an extension of our curiosity in the production of bioactive heterocycles (Mekky and Elwahy 2014; Salem et al. 2016, 2024; Mohamed et al. 2018, 2020; Kamel et al. 2022; Helmy et al. 2022; Fares et al. 2024, 2025; Ragheb et al. 2024), this study aimed to synthesize novel bis(cyanoacrylamide) derivatives incorporating piperazine and sulfamethoxazole moieties and evaluate their antimicrobial activity against *Aspergillus niger* and *Candida albicans*, *Enterococcus faecalis* ATCC 29212, *Staphylococcus aureus* ATCC 29213, *Pseudomonas aeuroginosa* ATCC 27853, *Escherichia coli* ATCC 25922, and *Klebsiella pneumoniae* ATCC 700603. Additionally, in silico molecular docking studies were conducted to assess the potential of the synthesized derivative as an inhibitor of bacterial and fungal proteins.

The overuse of antibiotics has led to the rise of drug-resistant bacteria, posing a severe threat to public health. Consequently, there is an urgent need for innovative treatments to target RA-associated bacteria and offer safer alternatives. 

## Materials and methods

### Materials

Melting points were determined on a Stuart melting point apparatus and are uncorrected. The IR spectra were measured as KBr pellets on an FTIR Bruker-Vector 22 spectrophotometer. ^1^H and ^13^C NMR spectra were recorded in DMSO-*d*_6_ as solvent at 300 MHz and 75 MHz, respectively, on a Varian Gemini NMR spectrometer using TMS as the internal standard. Mass spectra were measured on a Shimadzu GCMS-Q-1000 EX mass spectrometer at 70 eV. The elemental analyses were carried out at the Micro-Analytical Center, Cairo University, using an automated analyzer CHNS.

### Procedure

#### 2-Cyano-N-(4-{[(5-methylisoxazol-3-yl)amino]sulfonyl}phenyl)acetamide (3)

Sulphamethaxazole (10 mmol) **1** was added to 3-(1*H*-pyrazol-1-yl)−3-oxopropanenitrile **2** (10 mmol) in dry toluene (20 ml). The mixture was heated at reflux for 3 h. The solvent was evaporated, and the crude product was purified by crystallization from ethanol to give colorless crystals (90%). Mp 216–218 °C (Mohamed et al. 2020). IR (KBr): ν_max_/cm^−1^ 3331, 3279 (2NH), 2264 (C≡N), 1690 (C = O), 1397, 1167 (SO_2_). ^1^H NMR (400 MHz, DMSO-*d*_6_): *δ*/ppm 2.30 (s, 3H, C*H*_3_), 3.96 (s, 2H, C*H*_2_), 6.13 (s, 1H, isoxazole-C*H*), 7.73–7.75 (d, *J* = 8.8 Hz, 2H, Ar*H*), 7.82–7.85 (d, J = 8.8 Hz, 2H, Ar*H*), 10.71 (s, 1H, CON*H*), 11.37 (br, 1H, SO_2_N*H*). ^13^C NMR (100 MHz, DMSO-*d*_6_):* δ*/ppm 12.5, 27.5, 95.9, 116.1, 119.6, 128.7, 134.4, 143.1, 158.0, 162.5, 170.9. MS (EI, 70 eV): 320 [M^+^], Anal. Calcd. for C_13_H_12_N_4_O_4_S: C, 48.75; H, 3.78; N, 17.49. Found C, 48.46; H, 3.53; N, 17.28.

#### General procedure for the synthesis of 4,4'-((Piperazine-1,4-diylbis(2-oxoethane-2,1-diyl))bis(oxy))dibenzaldehyde 8 (Salem et al. 2022)

4-Hydroxybenzaldehyde **7** (10 mmol) was dissolved in hot ethanolic KOH solution (made by dissolving 0.56 g (10 mmol) of KOH in 10 ml of absolute ethanol), and the solvent was then removed in vacuo. The remaining material was dissolved in DMF (10 ml), and compound **6** (5 mmol) was added. The reaction mixture was refluxed for 10 min during which KCl was separated. The solvent was then evaporated under vacuum, and the remaining materials were poured onto crushed ice. The crude precipitate from ethanol/DMF to give **8**. Colorless solid (ethanol/acetic acid), (94% yield), mp. = 190–192 °C. IR (KBr, υ cm^−1^): 2865, 2744 (aldehyde CH), 1664 (CO). ^1^H NMR (300 MHz, DMSO-*d*_*6*_): δ 3.48–3.55 (m, 8H, -NCH_2_-), 5.04 (s, 4H, -OCH_2_-), 7.12 (d, 4H, Ar–H,* J* = 9 Hz),7.85 (d, 4H, Ar–H,* J* = 8.7 Hz), 9.87 (s, 2H, -CHO). ^13^C NMR (75 MHz, DMSO-*d*_*6*_): δ 43.9 (-NCH_2_-), 65.8, 115.2, 129.8, 131.6, 163.1 (ArC’s), 165.5 (CO–N), 191.3 (CHO). MS (EI, 70 eV): *m/z* 410 [M]^+^. Anal. Calcd. For C_22_H_22_N_2_O_6_: C, 64.38; H, 5.40; N, 6.83. Found: C, 64.37; H, 5.42; N, 6.87%.

#### General procedure for synthesis of (*E*)−2-cyano-3-(4-(2-(4-(2-(4-((*E*)−2-cyano-3-((4-(*N*-(5-methylisoxazol-3-yl)sulfamoyl)phenyl)amino)−3-oxoprop-1-en-1-yl)phenoxy)acetyl)piperazin-1-yl)−2-oxoethoxy)phenyl)-N-(4-(N-(5-methylisoxazol-3-yl)sulfamoyl)phenyl)acrylamide 9

A mixture of bis-aldehyde **8** (1 mmol) and compound **3** (2 mol) was heated at reflux in acetic acid (20 ml) containing sodium acetate (4 mmol) for 60 min. The crude product was collected and crystallized from EtOH.

Pale yellow solid (ethanol/DMF), (86% yield), > 300 °C; IR (KBr, υ cm^−1^): 3406 (NH), 2216 (CN), 1682 (CO). ^1^H NMR (300 MHz, DMSO) δ 2.29 (s, 6H, 2CH_3_), 3.50–3.57 (br s, 8H, piperazine-H), 5.05 (s, 2H, isoxazole-H4), 6.13 (s, 4H, -OCH_2_-), 7.15 (d, 2H, Ar–H, *J* = 9Hz), 7.86 (br, 4H, Ar–H), 7.99 (d, 2H, Ar–H, *J* = 9Hz), 8.24 (s, 2H, vinyl-H), 10.65 (br. s., 1H, 2NH), 11.68 (br. s., 1H, 2NH). ^13^C NMR (75 MHz, DMSO) δ 21.1, 44.0, 65.8, 95.5, 103.4, 115.7, 116.7, 120.4, 124.5, 128.0, 132.6, 134.2, 142.8, 151.3, 157.7, 161.6, 161.9, 165.6, 170.4, 172.2. MS (EI, 70 eV): *m/z* 1015 [M]^+^. Anal. Calcd. For C_48_H_42_N_10_O_12_S_2_: C, 56.80; H, 4.17; N, 13.80. Found: C, 56.62; H, 4.29; N, 13.66%.

### Bacterial and fungal species and culture condition

The tested bacterial species gram-positive bacteria including *Enterococcus faecalis* ATCC 29212 and *Staphylococcus aureus* ATCC 29213 and gram-negative bacteria such as *Pseudomonas aeuroginosa* ATCC 27853, *Escherichia coli* ATCC 25922, and *Klebsiella pneumoniae* ATCC 700603 were grown on a nutrient broth medium and incubated for 18 h at 37 °C (Techaoei et al. 2020). The fungal species including *Aspergillus niger* and *Candida albicans* were grown on Czapek–Dox broth medium and incubated for 48 h at 27 °C. The tested bacterial and fungal species were prepared as suspensions with a concentration equivalent to that of 0.5 McFarland Standard (Mohamed et al. 2012a).

### Antimicrobial activity

The disc-diffusion method was used to measure the antimicrobial activity of the synthesized compound. A sterile cotton swab was dipped into the adjusted suspension. The dried surface of the agar plates was inoculated by streaking the swab over the entire sterile agar surface and then allowed to dry for 15 min with the lid in place. The synthesized compound was tested at concentrations of 0.1, 0.2, 0.4, and 0.8 mg/ml against bacterial and fungal strains and dimethyl sulphoxide (DMSO) was used as a solvent. Blank Whatman filter paper disks with a diameter of 6 mm were impregnated with a tested dissolved compound in 10% DMSO. Bacterial strains were incubated at 37 °C for 24 h, while fungal species were incubated at 30 °C for 48 h. Tetracycline and fluconazole were used as positive standard controls for bacterial and fungal species, respectively, while DMSO was used as a negative control. The plates were kept at room temperature for 2 h before incubation. The plates were incubated at 37 °C for 24 h in the case of bacterial species and 28 °C for 48 h in the case of fungal species. After incubation, the antimicrobial activity was measured regarding the inhibition zone diameter in mm. This experiment was carried out in triplicate (Mohamed et al. 2012b; Hamed et al. 2020).

### Preparation of resazurin solution

In a sterile beaker, 337.5 mg of resazurin powder was dissolved in 50 ml of sterile distilled water to prepare the resazurin solution. The prepared solution was mixed for 1 h using a sterile vortex mixer to ensure homogeneity. The preparation steps were carried out in the dark, and the solution was then stored in a brown bottle since resazurin is light-sensitive (Teh et al. 2017).

### Measurement of minimum inhibitory concentration (MIC)

#### MIC of bacteria

In a 96-well microtiter plate, the tested compound was dissolved in nutrient broth (NB) at double the concentration of the final test. Column 1 contained 50 µl of the tested compound (320 mg/ml) with 50 µl of NB, while columns 2–5 included 50 µl of NB only. A micropipette was then used to transfer and mix the tested compound from columns 1–5, resulting in 50 µl tested compound per well. The different concentrations of the tested compound were obtained by double serial dilutions from columns 1 to 5. Lastly, 50 µl was removed from the fifth well and discarded. Column 6 contains the nutrient broth sterility and tested compound, while the serial dilution of antibiotic tetracycline (0.1 mg/ml) was performed from columns 8 to 12. The final concentration of the tested compound and tetracycline was now one-half of the original concentration in each well. Then, 5 µl of diluted bacterial suspension (1.5 × 10^6^ cell/ml) was added into all wells (except column 6, which contains the nutrient broth sterility and tested compound) and mixed thoroughly. After overnight incubation for 18–24 h at 37 °C, 5 µl resazurin (6.75 mg/ml) was added to all wells and incubated at 37 °C for 2–4 h. Changes of color were observed and recorded. On completion of the incubation, the MIC value is the lowest concentration of the tested compound with no color change (blue resazurin color remained unchanged) (Teh et al. 2017).

#### MIC of fungi

The MIC of the tested compound was determined by the agar dilution method. The stock solution of the synthesized compound was prepared in DMSO. Aliquots of the stock solution were used to prepare a series of subsequent concentrations (5, 10, 20, 40, 80, and 160 mg/ml). The plates were incubated at 27 °C for 7 days. Fluconazole was used with different concentrations (0.0625, 0.125, 0.25, 0.5, and 1 mg/ml) as a positive antibiotic. MIC of the tested compound was expressed as the lowest concentration (mg of the tested compound/ml of culture medium) at which no visible growth occurred compared with control after the incubation time.

### Protein preparation

The three-dimensional crystal structure of a secondary metabolism regulator laeA of *Aspergillus niger* (UniProt ID: G3XRG4), Agglutinin-like protein 3 of *Candida albicans* (UniProt ID: O74623), DNA gyrase subunit B of *Enterococcus faecalis* (UniProt ID: Q839Z1), Recombinase A (RecA) of *Escherichia coli* (UniProt ID: P0A7G6), cyclic AMP-AMP-AMP synthase of *Pseudomonas aeruginosa* (UniProt ID: P0DTF7), UDP-N-acetylglucosamine 1-carboxyvinyltransferase of *Klebsiella pneumoniae* (UniProt ID: A0A1S5RKE3), and clumping factor A of *Staphylococcus aureus* (UniProt ID: Q53653) structural protein were retrieved from the UniProt database and prepared using “prepare protein” protocol of BIOVIA Discovery Studio Visualizer 2021. Water molecules were removed, hydrogen atoms were added, and Gasteiger charges were assigned by using AutoDock tools (Morris et al. 2009). The binding sites of these proteins were predicted based on literature information and validated using the CB-DOCK2. The active sites identified were used for protein–ligand interaction studies (Liu et al. 2022). The grid boxes for docking were centered on the predicted binding sites. The exhaustiveness parameter was set to 8, and the default scoring function was used for the docking calculations (Trott and Olson 2010).

### Ligand preparation

The molecular structure of the tested compound was drawn using Marvin JS online free version and then extracted in SDF format, while tetracycline as a positive control of bacteria and fluconazole as a positive control of fungi were retrieved from PubChem database in SDF format. Avogdaro software (1.2.0) was used for energy minimization using MMFF94 force field to avoid steric overlap and to relax the conformation (Hanwell et al. 2012). These structures were then prepared using the ligand’s protocol of BIOVIA Discovery Studio Visualizer 2021.

### Molecular docking

Molecular docking is a method used to predict the binding orientation of small molecule candidates to their protein targets and, in turn, predict the binding affinity and strength of association between the target and ligand. The docking was performed between the active binding region of the structure of microbial protein and the tested compound using the Auto-Dock Vina 1.5.7 tool to predict the binding modes and affinities of the tested compound with proteins of microbial species (Baby et al. 2016).

### Visualization and analysis

The protein–ligand interactions were visualized and analyzed using BIOVIA Discovery Studio Visualizer 2021 (Fakih and Dewi 2021). The binding affinities (ΔG values) and intermolecular interactions, including hydrogen bonds and hydrophobic interactions, were analyzed and reported. The binding affinities (ΔG values) of the ligand with a unit of kcal/mol were noted as a negative ranking.

### Statistical analysis

The values of the antimicrobial activity of the tested compound were expressed as the mean ± standard deviation of triplicate for each sample. This analysis was performed by using SPSS (version 21), where one-way ANOVA was used followed by the Duncan test with a significant level (*P* < 0.05) (Colao et al. 2005).

## Results and discussion

### Chemistry

**Scheme 1:**
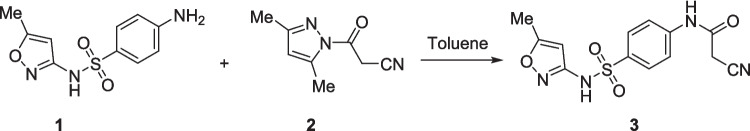
Synthesis of 2-cyano-*N*-(4-(*N*-(5-methylisoxazol-3-yl)sulfamoyl)phenyl)acetamide **3**

The precursor, 2-cyano-*N*-(4-(*N*-(5-methylisoxazol-3-yl)sulfamoyl)phenyl)acetamide **3** that was chosen for this synthesis was produced via the reaction of sulphamethoxazole **1** with 1-cyanoacetylpyrazole **2** following the described protocols (Scheme 1) (Štetinová et al. 1996; Gorobets et al. 2004; Mohamed et al. 2017b, a).

**Scheme 2:**
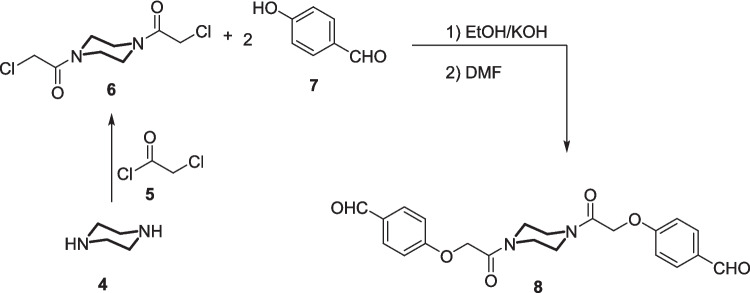
Synthesis of bis-benzaldehyde-based-piperazine **8**

Moreover, the bis-benzaldehyde-based-piperazine **8** was prepared in 90% yield by reacting the potassium salt of the 4-hydroxybenzaldehyde **7** with 1,1′-(piperazine-1,4-diyl)bis(2-chloroethan-1-one) **6** (prepared by stirring piperazine **4** with two equivalents of 2-chloroacetyl chloride **2**) in boiling DMF (Scheme 2).

**Scheme 3:**
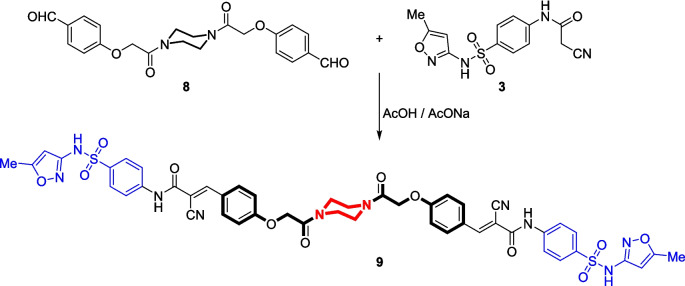
Synthesis of bis-cyanoacrylamide-based piperazine and incorporating sulphamethoxazole moiety

With the required starting materials in hand, we were able to synthesize the target compound, bis-cyanoacrylamide **9**, incorporating sulphamethoxazole and bearing the piperazine core via phenoxymethyl linker. Thus, Knoevenagel condensation reaction of bis(aldehyde) **8** with the cyanoacetylsulphamethoxazole **3** in acetic acid in the presence of sodium acetate as basic catalyst resulted in the formation of bis(cyanoacrylamide) **9** (Scheme 3).

Spectral data analysis validated the structure of compound **9**. Mass spectrum revealed the molecular ion peak at *m/z* 1015. The constitution of **9** was also supported based on the IR spectrum that indicated a broad band at 3406 for the NH group and a band at 2216 for the cyano group. Besides, it revealed two carbonyl groups at 1682 cm^−1^. ^1^H NMR revealed a singlet signal at *δ* 2.29 ppm for the 5-methylisoxazole. Additionally, it displayed two singlet signals at *δ* 5.05 and 8.24 for the respective isoxazole-H4 and vinyl-H3 protons. The amide and sulphonamide NH groups showed large signals at *δ* 10.65 and 11.68. Aromatic proton signals occur as two doublets and in the range of 7.15–7.99 ppm besides broad signal at 7.86 ppm. Additionally, the ^13^C-NMR revealed 19 signals related to 19 different carbons.

### Effect of the tested compound on the growth of the different fungal and bacterial species

**Chart 1:**
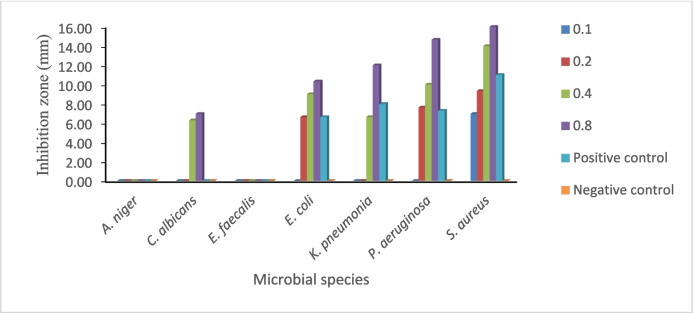
Antimicrobial activity represented by inhibition zone (mm) of the tested compound at concentrations of 0.1, 0.2, 0.4, and 0.8 mg/ml. The positive control (tetracycline and fluconazole) was used at 0.1 mg/ml

The tested compound at the concentrations of 0.1, 0.2, 0.4, and 0.8 mg/ml exhibited antifungal activities against *Aspergillus niger* and *Candida albicans* and antibacterial activities against *Enterococcus faecalis* ATCC 29212, *Escherichia coli* ATCC 25922, *Klebsiella pneumonia* ATCC 700603, *Pseudomonas aeruginosa* ATCC 27853, and *Staphylococcus aureus* ATCC 29213 as shown in Table 1 and Chart 1. Furthermore, tetracycline and fluconazole were used as standards for antibacterial and antifungal activity, respectively. The antibacterial and antifungal activities of the tested compound increased with increasing concentration. The antimicrobial activity of the tested compound may be due to the presence of piperazine as recorded by Jalageri et al. (2021) who stated that piperazine may cause cell lysis, disrupt cellular functions, and alter membrane integrity. Piperazine has been used to create highly effective sixth-generation antibiotics and is considered a valuable starting point for developing new drugs. Piperazine provides a large polar surface area, structural rigidity, and hydrogen-bond acceptors and donors, which often lead to enhanced target affinity, specificity, water solubility, oral bioavailability, and ADME (i.e., absorption, distribution, metabolism, and excretion) properties. The exitance of a nitro group in the piperazine structure is frequently linked to its antimicrobial properties (Mary et al. 2021).

**Table 1:** Antimicrobial activity represented by inhibition zone (mm) of the tested compound at concentrations of 0.1, 0.2, 0.4, and 0.8 mg/ml

Microbial species	Compound (mg/ml)				Tetracycline	Fluconazole	Negative control
	0.1	0.2	0.4	0.8			
*Aspergillus niger*	0	0^a^	0^a^	0^a^	0^a^	0^a^	0^a^
*Candida albicans*	0^a^	0^a^	6.33^b^ ± 0.58	7.0^b^ ± 1.0	0^a^	0^a^	0^a^
*Enterococcus faecalis*	0^a^	0^a^	0^a^	0^a^	0^a^	0^a^	0^a^
*Echerichia coli*	0^a^	6.67^b^ ± 0.58	9^c^ ± 0.00	10.33^bc^ ± 0.57	6.67^b^ ± 1.15	0^a^	0^a^
*Klebsiella pneumonia*	0^a^	0^a^	6.67^b^ ± 1.15	12.0^ cd^ ± 3.0	8.0^b^ ± 3.46	0^a^	0^a^
*Pseudomonas aeruginosa*	0^a^	7.66^b^ ± 2.08	10.0^c^ ± 2.64	14.67^d^ ± 5.03	7.33^b^ ± 1.53	0^a^	0^a^
*Staphylococcus aureus*	7.0^b^ ± 1	9.33^c^ ± 0.58	14^d^ ± 1.0	16.0^d^ ± 1.0	11.0^c^ ± 1.0	0^a^	0^a^
LSD (0.05)	1.15	1.88	2.27	2.96	2.08	0	0

Numbers expressed as mean ± standard deviation (*n* = 3) for each sample. Different small letters in the same column show mean values at a significant level (*p* < 0.05). The positive control (tetracycline and fluconazole) was used at 0.1 mg/ml

Significant antibacterial activity was shown by the tested compound at a concentration of 0.8 mg/ml, which led to the inhibition of *E. coli*, *K. pneumonia*, *P. aeruginosa*, and *S. aureus* with inhibition zones of 10.33 mm, 12 mm, 14.67 mm, and 16 mm, respectively. The observed variation in antimicrobial activity among different bacterial species is expected, as gram-positive bacteria, such as *Staphylococcus aureus*, are generally more susceptible to antimicrobial agents than gram-negative bacteria. This difference in susceptibility is primarily due to the structural differences in their cell walls (Bąchor et al. 2023). Piperazine-methoxyphenyl polymer exhibits promising antibacterial effects by inhibiting bacterial DNA gyrase B, β-ketoacyl-ACP synthase III, enoyl reductase, and glycosyltransferase (Fares et al. 2024). Likewise, the presence of methyl isoxazole in the tested compound can increase its inhibition activity. Methyl isoxazole can damage the microbial cell wall or membrane, leading to leakage of cell contents and subsequent cellular damage. This effect is likely due to the positive charge of sulfur atoms in the chemical structure (Mary et al. 2021). Hawash et al. (2022) discussed that compound *N*-(4-(*tert*-butyl)phenyl)−3-(4-fluorophenyl)−5-methylisoxazole-4-carboxamide showed potent inhibitory activity and antioxidant activity, capable of suppressing the activity of lipase and amylase enzymes. The presence of sulfur causes bacterial cell damage as recorded by Ragab et al. (2021) who found that sulfonyl derivatives inhibited the growth of *E. coli*, *C. albicans*, *and S. aureus* due to their bacteriostatic properties, which increased cell lysis, shrinkage, and loss of confluency.

The tested compound presented efficient growth inhibition of *C. albicans* with inhibition zones of 7 mm, at a concentration of 0.8 (mg/ml) as compared to fluconazole, a positive control for the tested fungi, which showed no inhibition activity. Piperazine-containing ciprofloxacin dimers also showed significant antifungal activity as these compounds exhibit favorable oral bioavailability and membrane permeability, typically showing high absorption in the gastrointestinal tract (Sroor et al. 2021). Acrylamide derivatives have antifungal properties that target ergosterol, which is a vital component of fungal cell membranes. Binding acrylamide to ergosterol disrupts membrane fluidity and cellular functions leading to the formation of holes that damage the cell membrane and cause fungal death (Zhang et al. 2015). Additionally, acrylamide derivatives showed moderate to strong inhibition of DNA gyrase and dihydrofolate reductase enzymes (Sutton et al. 2021). Lee et al. (2023) and Torres-Figueroa et al. (2020) indicated that acrylamide derivatives exhibited antibacterial against *E. coli* and *Scaphirhynchus albus* and antifungal activities due to the electrostatic attraction and hydrophobic functionality, while ionic bonding may aid in the initial interaction between the derivative and the cell. The hydrophobic substituents contribute to being primarily responsible for disrupting the bacterial lipid membrane.

The resistance of *Enterococcus faecalis* to the tested compound may be due to biofilm formation abilities. Biofilm mechanisms protect against various environmental stressors, including chemical (antimicrobial agents), physical (UV light and dehydration), and biological (immune cells) ones. Both abiotic and biotic surfaces can support the development of biofilms (Ren et al. 2024). However, the presence of efflux pumps such as EmeA, bcr, AcrAB, and mdtABC genes in *E. faecalis* can contribute resistance by actively pumping out the antimicrobial agents (Bąchor et al. 2023; De Gaetano et al. 2023). Quorum sensing is a technique used by microbes to perform cell–cell communications through autoinducers which is a chemical signal released by microbe. Bacteria produce signal molecules called autoinducers that are capable of communicating and controlling processes like biofilm formation and antibiotic resistance. The use of molecules to confuse bacterial contact and virulence may therefore lead to frustrating bacterial tolerance and to provide a successful conventional treatment (El-Khouly et al. 2021).

### The MIC determined for the fungal and bacterial species tested

The MICs of the tested compound on fungal and bacterial species are presented in Table 2. *Escherichia coli* ATCC 25922 and *Pseudomonas aeruginosa* ATCC 27853 were the most susceptible species to the lowest concentration of the tested compound with MIC = 5 mg/ml, followed by *Klebsiella pneumonia* ATCC 700603 (MIC = 10 mg/ml). Gram-negative bacterial pathogens were more susceptible to the tested compound compared to gram-positive bacterial pathogens, which may be due to the thick peptidoglycan layer in the cell wall of gram-positive bacteria. Gram-positive bacterial strains are generally composed of a three-dimensional thick peptidoglycan layer that possesses linear polysaccharide chains cross-linked by more short peptides and thus forms a complex structure compared to that of gram-negative bacteria (Hamed et al. 2020). A series of piperazine derivatives showed antimicrobial activity against *Bacillus subtilis*, *Staphylococcus epidermidis*, *Escherichia coli*, *Pseudomonas aeroginosa*, *Candida albicans*, and *Aspergillus niger* reflecting moderate to good activity with minimum inhibitory concentration of 0.0248 µmol/ml (Yadav et al. 2022). Piperazine derivatives showed excellent antimicrobial efficacy against *Pseudomonas aeruginosa*, *Staphylococcus aureus*, *Escherichia coli*, *Aspergillus niger*, and *Candida albicans* with MICs of 50 μg/ml (Desai et al. 2022).

**Table 2:** Minimum inhibitory concentrations (MIC) (mg/ml) of the tested compound on the bacterial and fungal species

Microbial species	Compound (mg/ml)	Tetracycline	Fluconazole
*Aspergillus niger*	> 160	-	0.08
*Candida albicans*	160	-	> 1000
*Enterococcus faecalis*	40	0.05	-
*Escherichia coli*	5	0.1	-
*Klebsiella pneumonia*	10	0.05	-
*Pseudomonas aeruginosa*	5	0.05	-
*Staphylococcus aureus*	40	0.1	-

The tested compound showed complete inhibition of gram-positive bacteria *Enterococcus faecalis* ATCC 29212 and *Staphylococcus aureus* ATCC 29213 at a concentration of 40 mg/ml, while *C. albicans* was completely inhibited at a concentration of 160 mg/ml. The methylisoxazole in the tested compound confirmed antibacterial activity against *Staphylococcus aureus*, potentially leading to growth inhibition and cell death (Sroor et al. 2021), while acrylamide targets the exposed layer of the peptidoglycan wall in gram-positive bacteria *S. aureus* (Bernardi et al. 2021)*.* The MIC of pyridazine, pyrazolines, thiazole, pyrimidine, and thiazine derivatives containing a sulfonamide group against *C. albicans*, *E. coli*, and *S. aureus* ranged from 200 to 1000 μg/ml, demonstrating their potential as promising antimicrobial agents (Ziwar 2024).

*Aspergillus niger* was the most resistant species to the tested compound, which showed no antifungal activity. The resistance of *A. niger* may be due to different mechanisms as recorded by Namaki Kheljan et al. (2022) who explained that *A. niger* may develop resistance to antifungal drugs through several mechanisms. One possibility is that the fungus could increase the efflux of the drug from its cells, reducing its intracellular concentration. Additionally, *A. niger* might activate cellular stress responses to mitigate the effects of the antifungal agent. Epigenetic regulation of *A. niger* may also increase resistance by DNA methylation, histone modifications, and non-coding RNAs, which effect on fungal growth, survival, and pathogenicity (Sreekantan et al. 2022).

### Molecular docking

Modeling studies were used to visualize the binding model and interaction of the tested compound within the active site of the target protein. Accordingly, molecular docking studies were performed against five bacterial proteins and two fungal proteins, including DNA gyrase subunit B (UniProt ID: Q839Z1), protein RecA of (UniProt ID: P0A7G6), cyclic AMP-AMP-AMP synthase (UniProt ID: P0DTF7), UDP-N-acetylglucosamine 1-carboxyvinyl transferase (UniProt ID: A0A1S5RKE3), and clumping factor A (UniProt ID: Q53653), which are expressed for *Enterococcus faecalis*, *Escherichia coli*, *Pseudomonas aeruginosa*, *Klebsiella pneumoniae*, and *Staphylococcus aureus*, respectively*.* However, secondary metabolism regulator laeA (UniProt ID: G3XRG4) and agglutinin-like protein 3 (UniProt ID: O74623) are expressed for *Aspergillus niger* and *Candida albicans*, respectively. Proteins were optimized by removing water molecules, adding polar hydrogens, and assigning Gasteiger charges. The bond order of the ligand and protein was adjusted. Target compounds were built using the Marvin JS online and then subjected to energy minimization by Avogadro software.

**Fig. 1:**
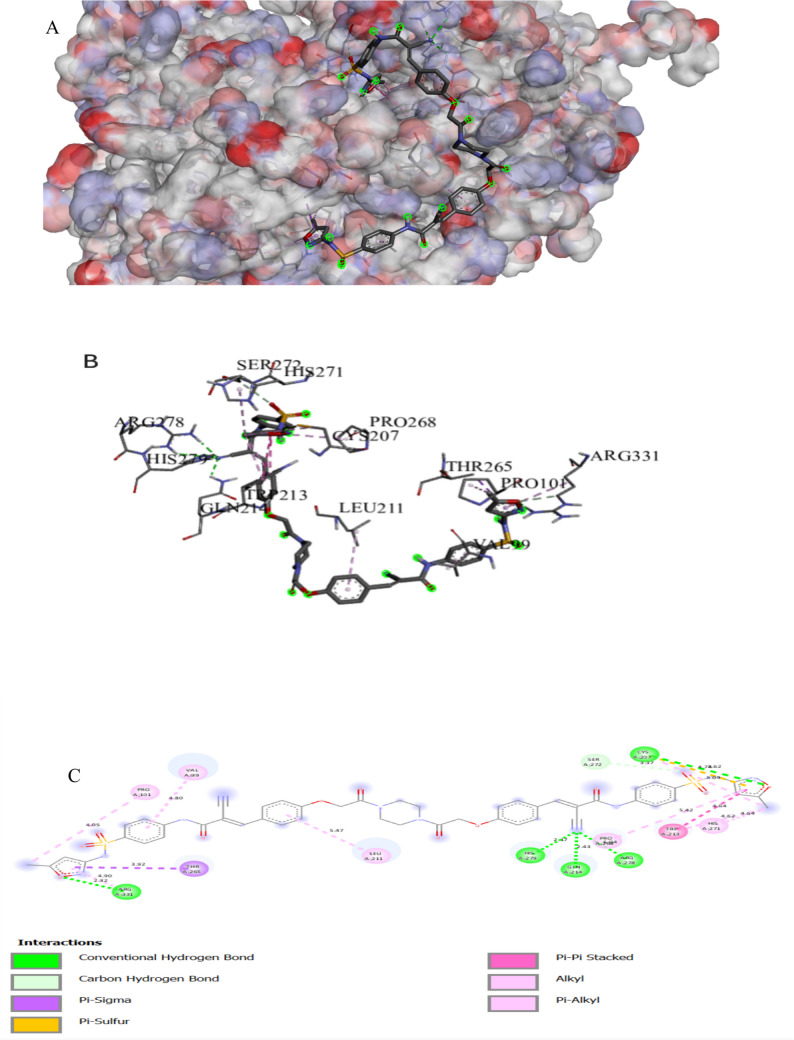
Protein–ligand complex of the tested compound with the active site of secondary metabolism regulator laeA in *Aspergillus niger* (UniProt ID: G3XRG4) (**A** and **B**) and 2D interaction of the protein with the compound (**C**)

**Fig. 2:**
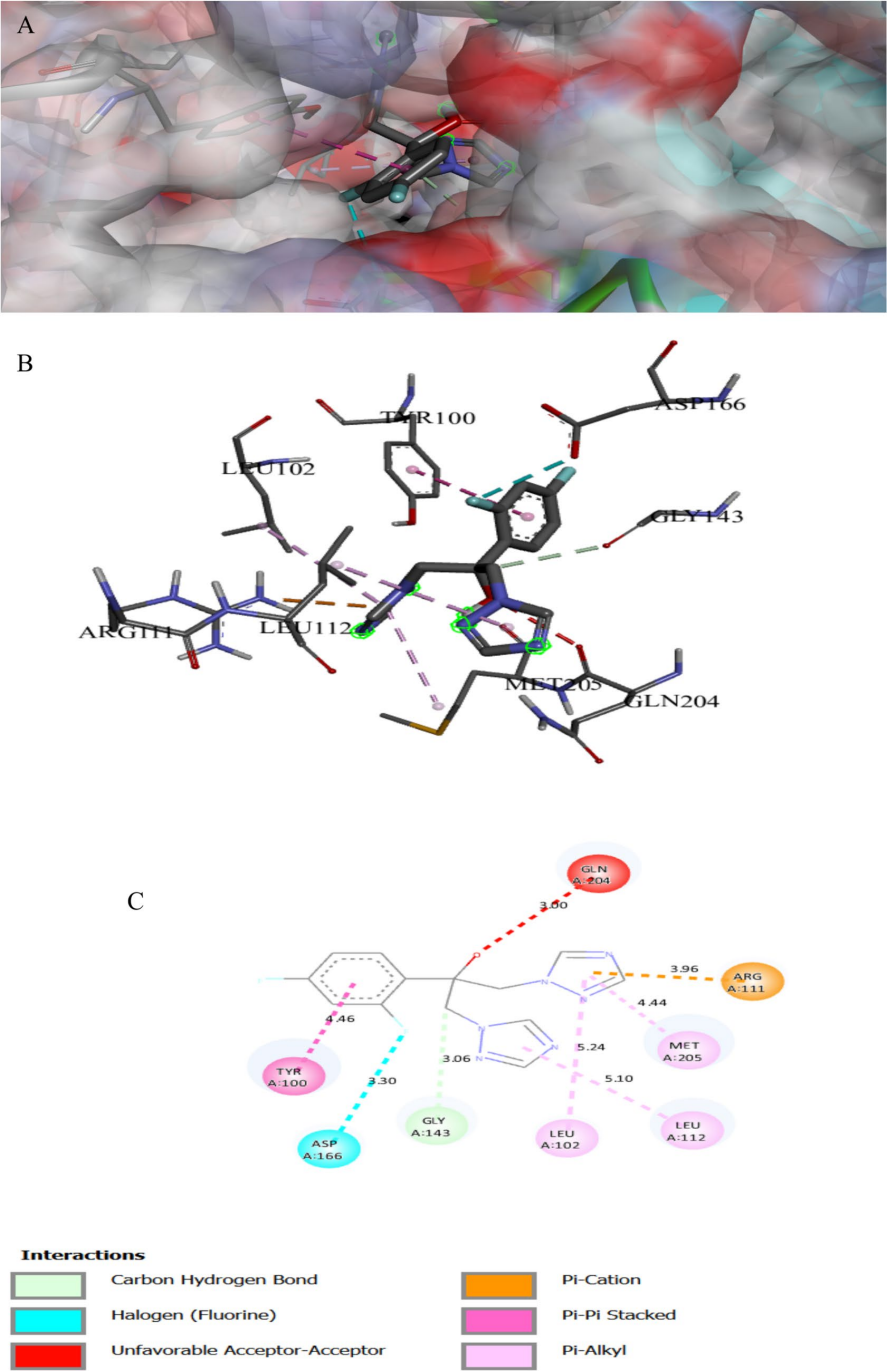
Protein–ligand complex of the fluconazole with the active site of secondary metabolism regulator laeA in *Aspergillus niger* (UniProt ID: G3XRG4) (**A** and **B**) and 2D interaction of the protein with fluconazole (**C**)

The binding interactions of the tested compound and fluconazole with the active site of the secondary metabolism regulator laeA in *A. niger* (UniProt ID: G3XRG4) are shown in Figs. 1 and 2. LaeA controls the gene expression of the citric acid synthesis enzyme in *A. niger*, which is essential for citric acid production (Niu et al. 2016). LaeA can regulate development and secondary metabolism in many fungal species. Additionally, LaeA also plays an important role in regulating OTA (Ochratoxin A) biosynthesis in *A. niger* (Zhang et al. 2022). The tested compound interacted with the LaeA active site, exhibiting a binding score of ∆G =  − 8 kcal/mol, which is less negative compared to the binding score of fluconazole (∆G =  − 7 kcal/mol). The tested compound bonded tightly to the LaeA active site through 16 interactions, four of them were conventional H bonds, three carbon, three Pi-alkyl, three alkyl, one pi-anion, two pi-cation, one pi-sigma, one halogen, and one pi pi-stacked interaction, while fluconazole exhibited only seven interactions with the LaeA active sites (Table 3). The presence of sulfonamide and azomethine groups and some heteroaryl rings in an individual molecule could have increased the antibacterial actions, while the antifungal action could be attributed to the presence of guanyl and acetyl groups in the molecular structure. The sulphonyl compound makes a hydrogen-bonding interaction that mediates between the oxygen of the sulphonyl group and the side chain of SER272 (Bishoyi et al. 2022).

**Fig. 3:**
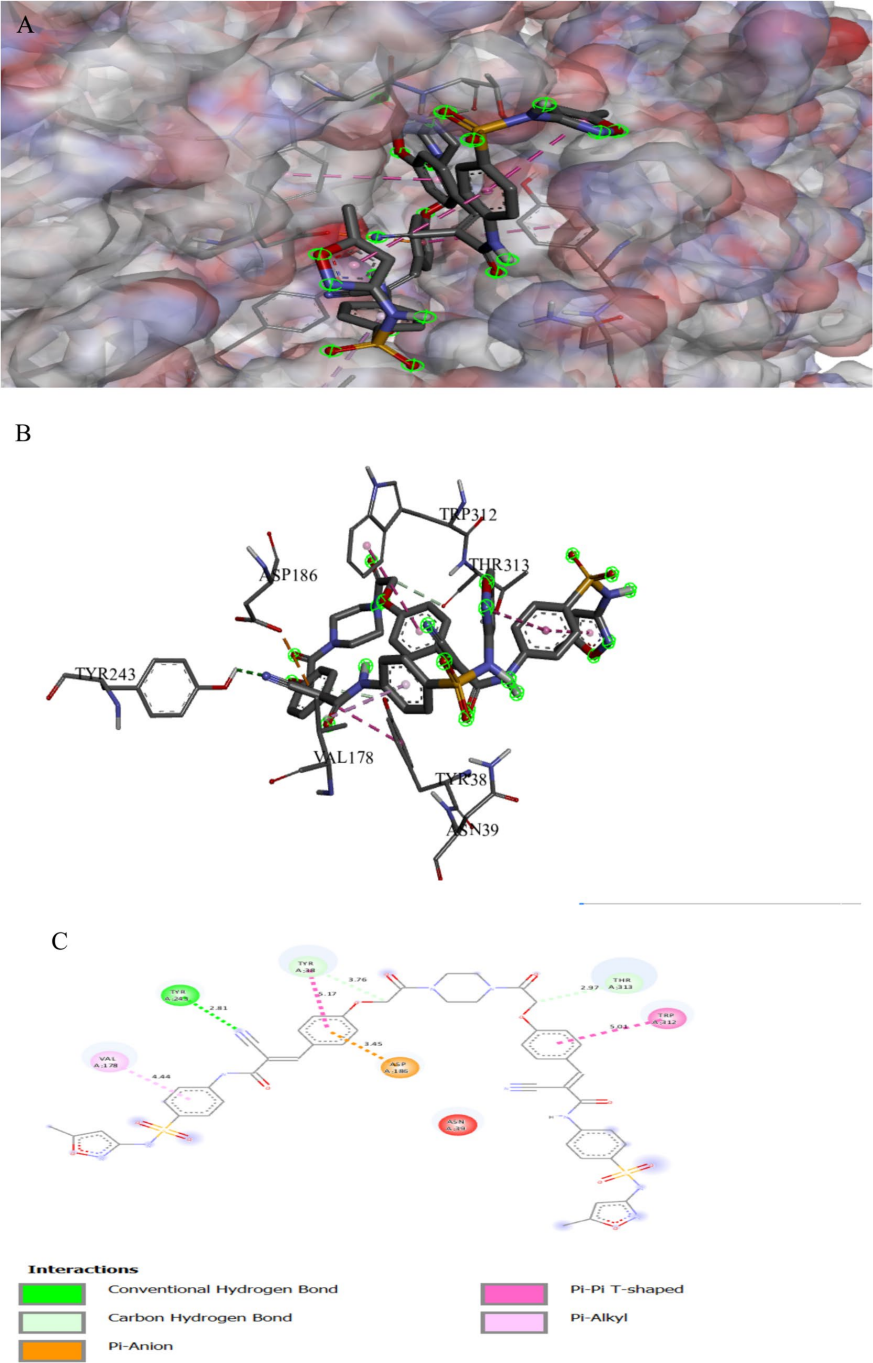
Protein–ligand complex of the tested compound with the active site of Agglutinin-like protein 3 in *Candida albicans* (UniProt ID: O74623) (**A** and **B**) and 2D interaction of the protein with the compound (**C**)

**Fig. 4:**
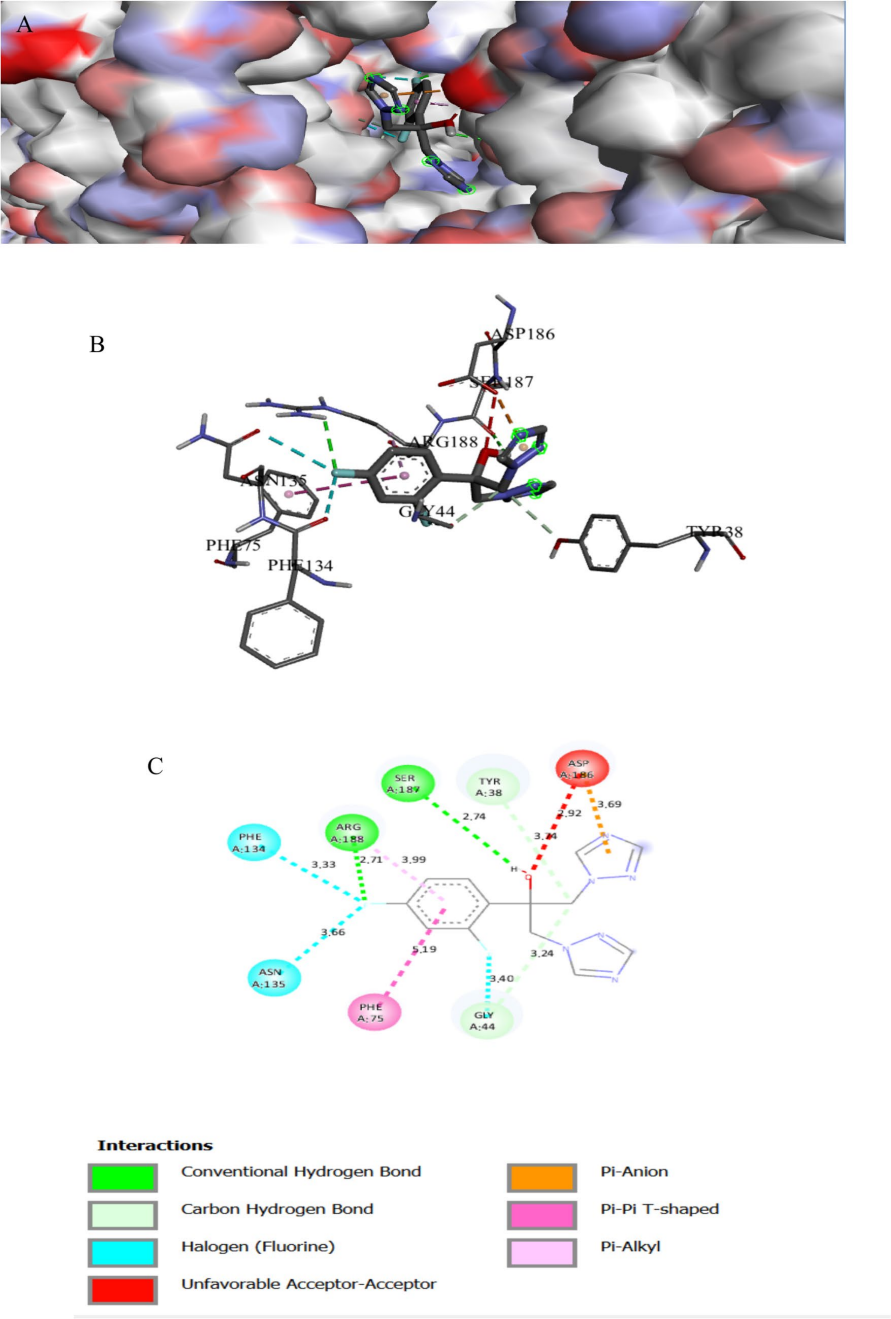
Protein–ligand complex of fluconazole with the active site of Agglutinin-like protein 3 in *Candida albicans* (UniProt ID: O74623) (**A** and **B**) and 2D interaction of the protein with fluconazole (**C**)

Figures 3 and 4 illustrate the binding interactions of the tested compound and fluconazole with the active site of the agglutinin-like protein 3 (Als3) in *C. albicans* (UniProt ID: O74623). Als3 plays an essential role in adherence and biofilm formation, contributing to the antifungal resistance of *C. albicans*. Als3 produced a dense biofilm structure, making it harder for the antifungal drugs to reach the inner part of the biofilms and finally increase the antifungal resistance (Liu et al. 2021). The tested compound showed an interaction with the agglutinin-like protein 3 active site, achieving a binding score of ∆G =  − 9.2 kcal/mol, which is less negative than that of a binding score of fluconazole (∆G =  − 8.3 kcal/mol). The tested compound bonded tightly to the Als3 active site through 10 interactions, two of them were carbon bonds, one Pi-alkyl, three alkyls, one pi-anion, one pi-sulfonyl group, one pi-sigma, and one pi pi-stacked interaction, while fluconazole showed 10 interactions with Als3 active sites. The tested compound binds to protein residues of PRO46, THR45, ASP186, LEU310, TRP312, VAL178, ARG188, PHE75, and TYR183, while fluconazole binds to amino acids of ARG188, SER187, GLY44, PHE134, GLY44, ASN135, ASP186, TYR38, ARG188, and PHE75 (Table 4). Specifically, the sulfonamide derivative compound exhibited the formation of a hydrogen bond with *C. albicans* as recorded by Kapısuz et al. (2024) who found five conventional hydrogen bonds with the amino acid THR312.

**Fig. 5:**
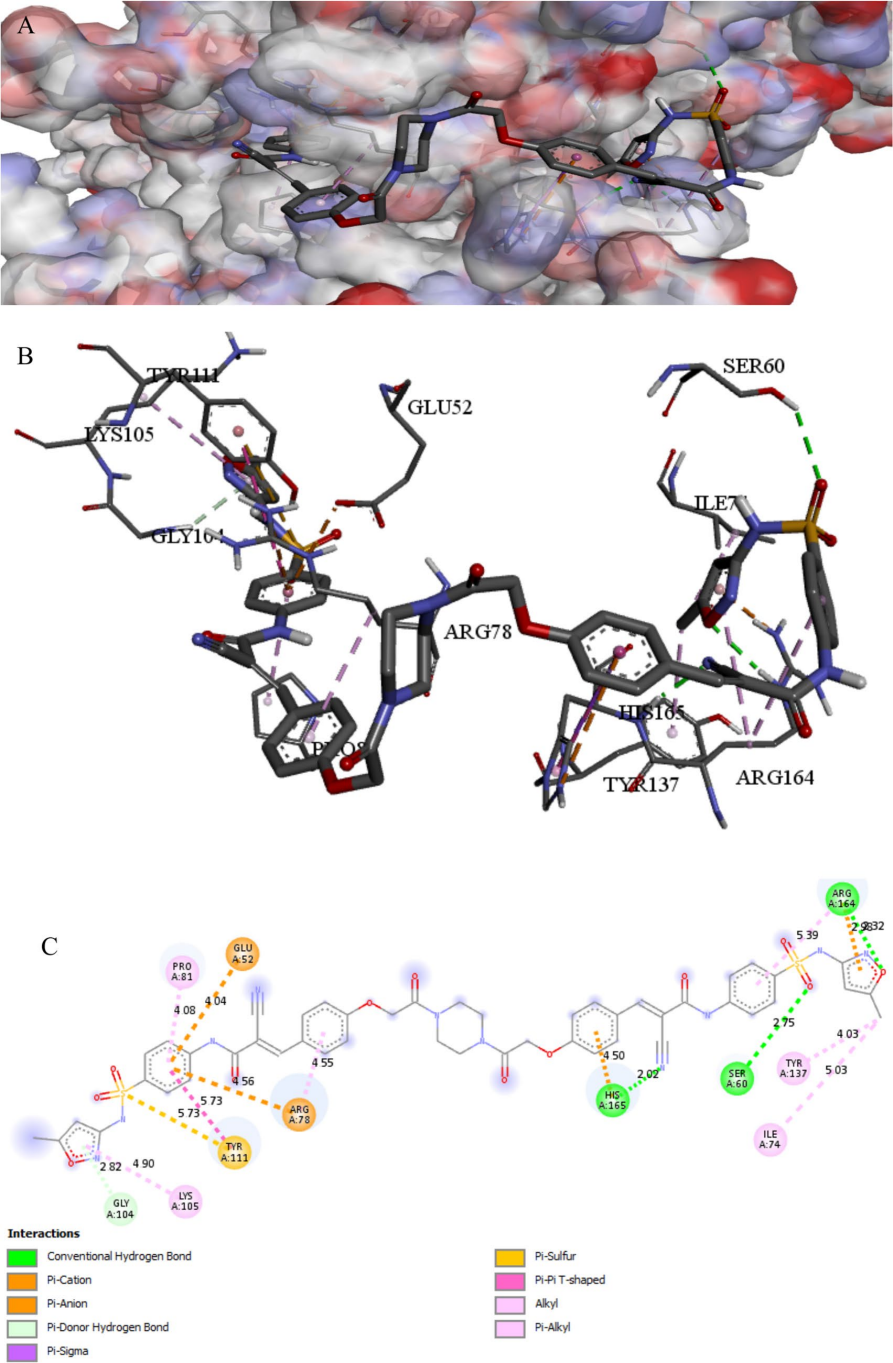
Protein–ligand complex of the tested compound with the active site of DNA gyrase subunit B in *Enterococcus faecalis* (UniProt ID: Q839Z1) (**A** and **B**) and 2D interaction of the protein with the compound (**C**)

**Fig. 6:**
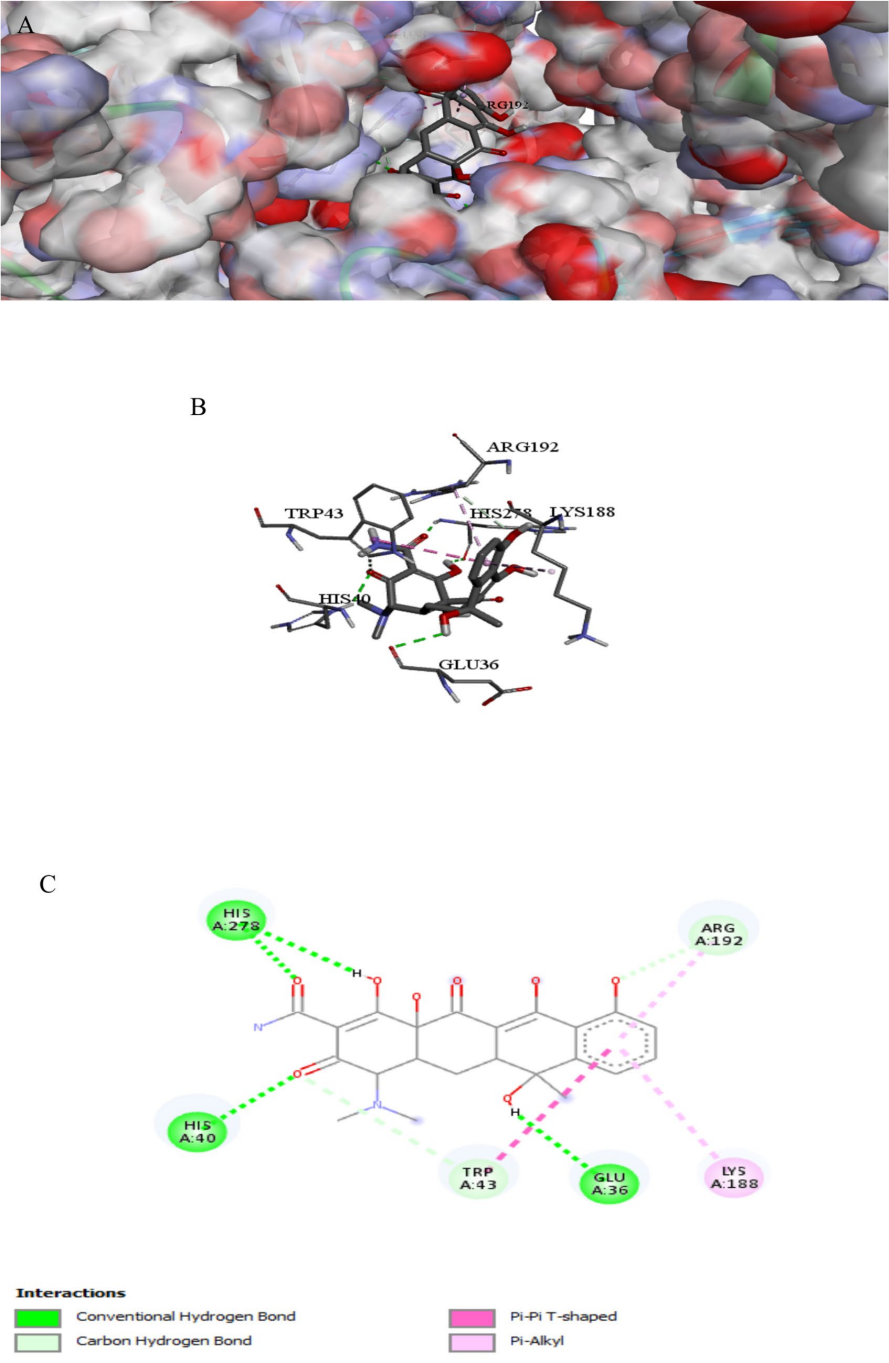
Protein–ligand complex of the tetracycline with the active site of DNA gyrase subunit B in *Enterococcus faecalis* (UniProt ID: Q839Z1) (**A** and **B**) and 2D interaction of the protein with tetracycline (**C**)

The binding interactions of the tested compound and tetracycline with the active site of the DNA gyrase subunit B in *Enterococcus faecalis* (UniProt ID: Q839Z1) are shown in Figs. 5 and 6. The tested compound showed an interaction with the DNA gyrase subunit B active site, achieving a binding score of ∆G =  − 7.0 kcal/mol, which is less negative than that of tetracycline (∆G =  − 9 kcal/mol). ATP-dependent bacterial GyrB enzyme is crucial for maintaining DNA topology and ensuring bacterial cell survival. Bacterial DNA GyrB is also a validated target for the development of new antibacterial agents (Gurram and Azam 2021). The tested compound bonded tightly to the active site through 3 interactions, one of them was conventional H bonds, one Pi-alkyl, and one pi-sigma, while tetracycline showed only six interactions with active sites (Table 5). The piperazine derivative compound makes hydrogen bonding interactions with hydrogen atoms of GLY109 (Patil et al. 2021).

**Fig. 7:**
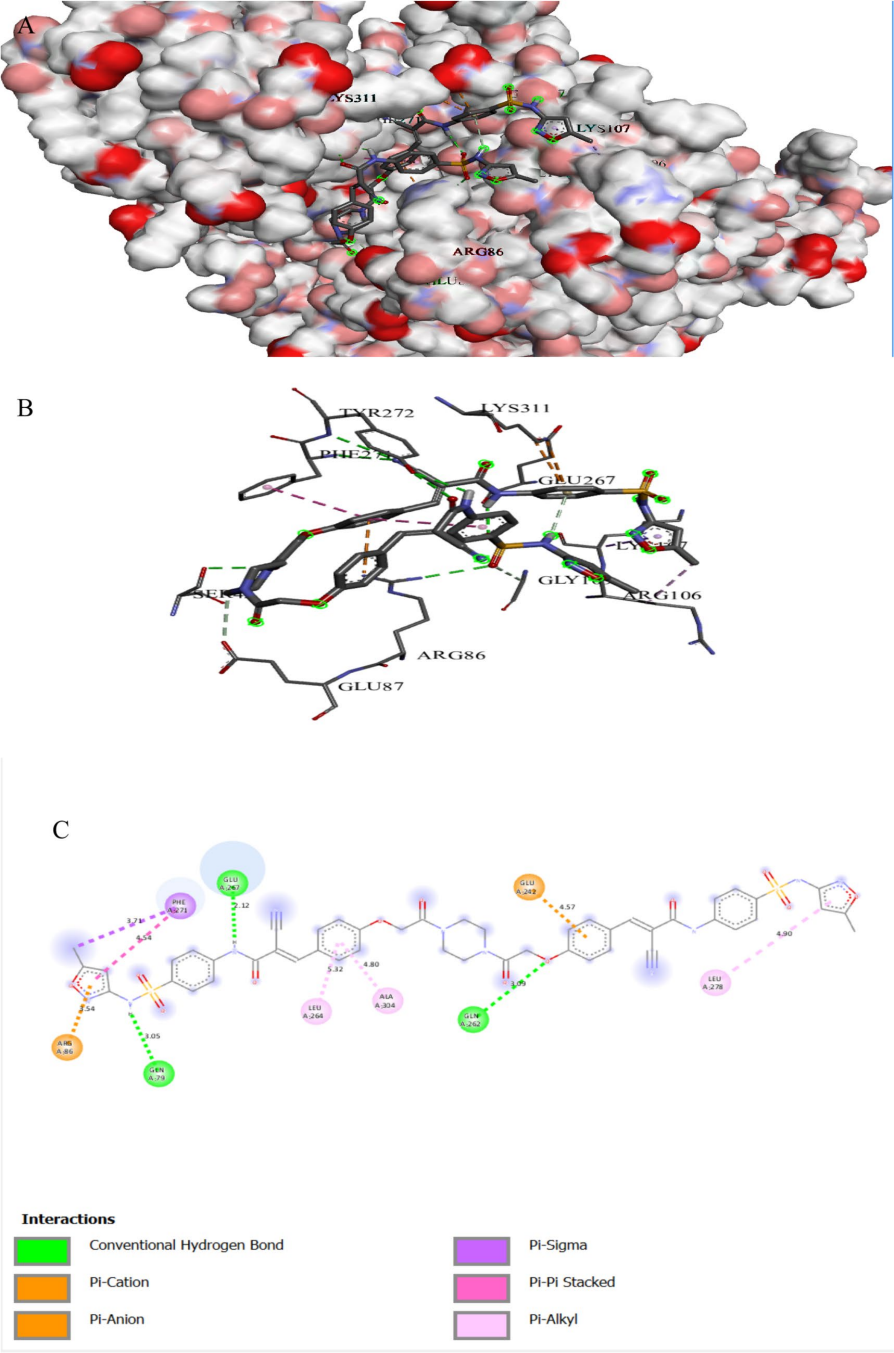
Protein–ligand complex of the tested compound with the active site of protein RecA of *Escherichia coli* (UniProt ID: P0A7G6) (**A** and **B**) and 2D interaction of the protein with the compound (**C**)

**Fig. 8:**
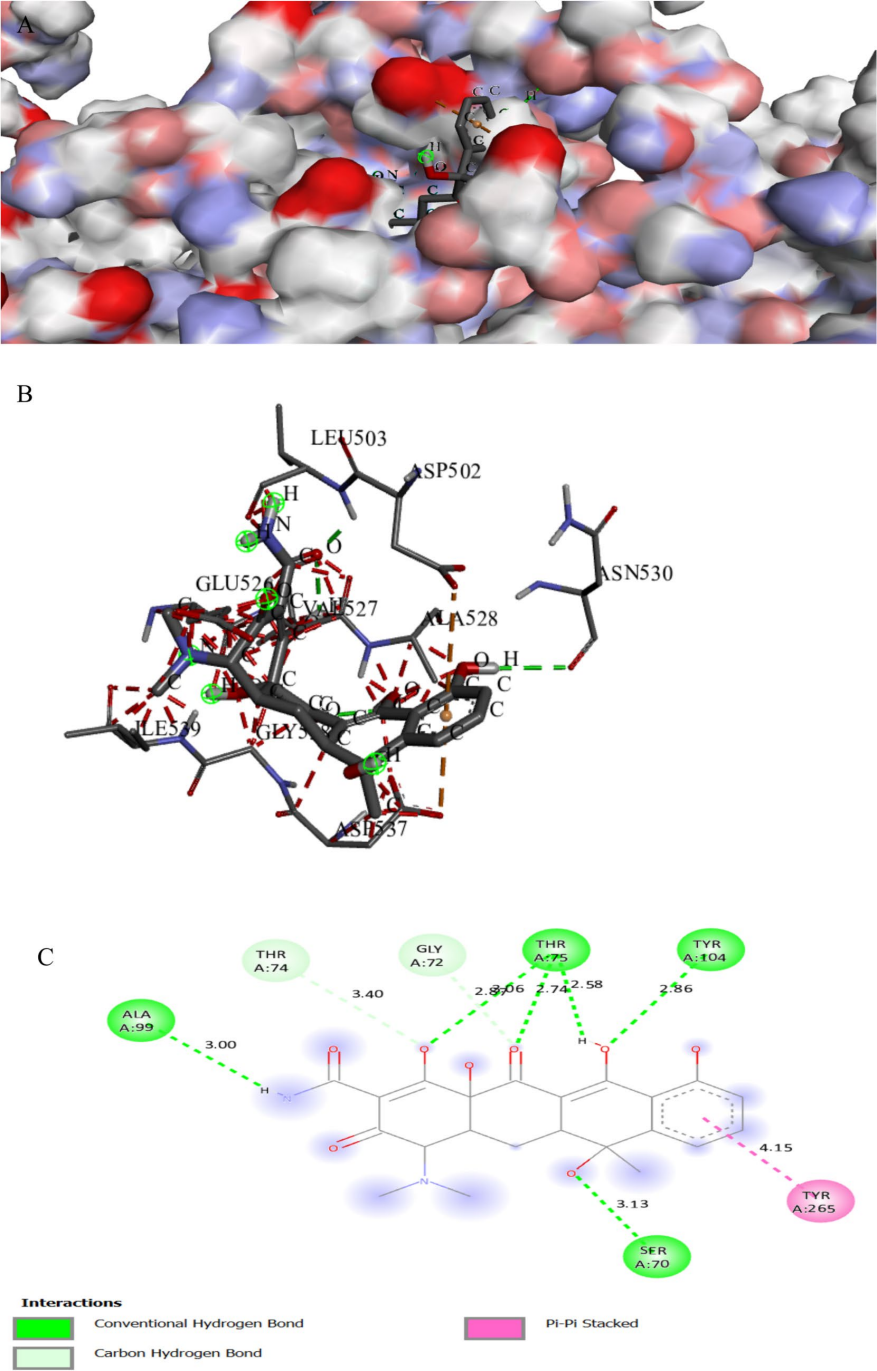
Protein–ligand complex of tetracycline with the active site of protein RecA of *Escherichia coli* (UniProt ID: P0A7G6) (**A** and **B**) and 2D interaction of the protein with tetracycline (**C**)

Figures 7 and 8 show the binding mode of interaction between the tested compound and tetracycline with the Recombinase A (RecA) active site in *Escherichia coli* (UniProt ID: P0A7G6). The tested compound binds to the active site of RecA which may affect the SOS response, which is a bacterial stress response mechanism activated when bacteria encounter DNA damage that helps bacteria survive under stress and can contribute to the development of antibiotic resistance. SOS inhibition could strengthen the antibiotic. RecA L2 is characterized by its role in filamentation and homologous recombination (Cory et al. 2023). The tested compound interacted with the active site of protein RecA with a binding score of ∆G =  − 8.5 kcal/mol, which is less negative than a binding score of the tetracycline (∆G =  − 7.7 kcal/mol). The tested compound bonded tightly to the active site through 10 interactions, four of them were conventional H bonds, two Pi-alkyl, two pi-anion, one pi-cation, one pi-sigma, and one pi pi-stacked, while tetracycline showed nine interactions with active sites (Table 6). The piperazine-derived molecule forms hydrogen bonds between the hydrogen atoms of LYS281 and the oxygen atom of the piperazine carbonyl group (Patil et al. 2021).

**Fig. 9:**
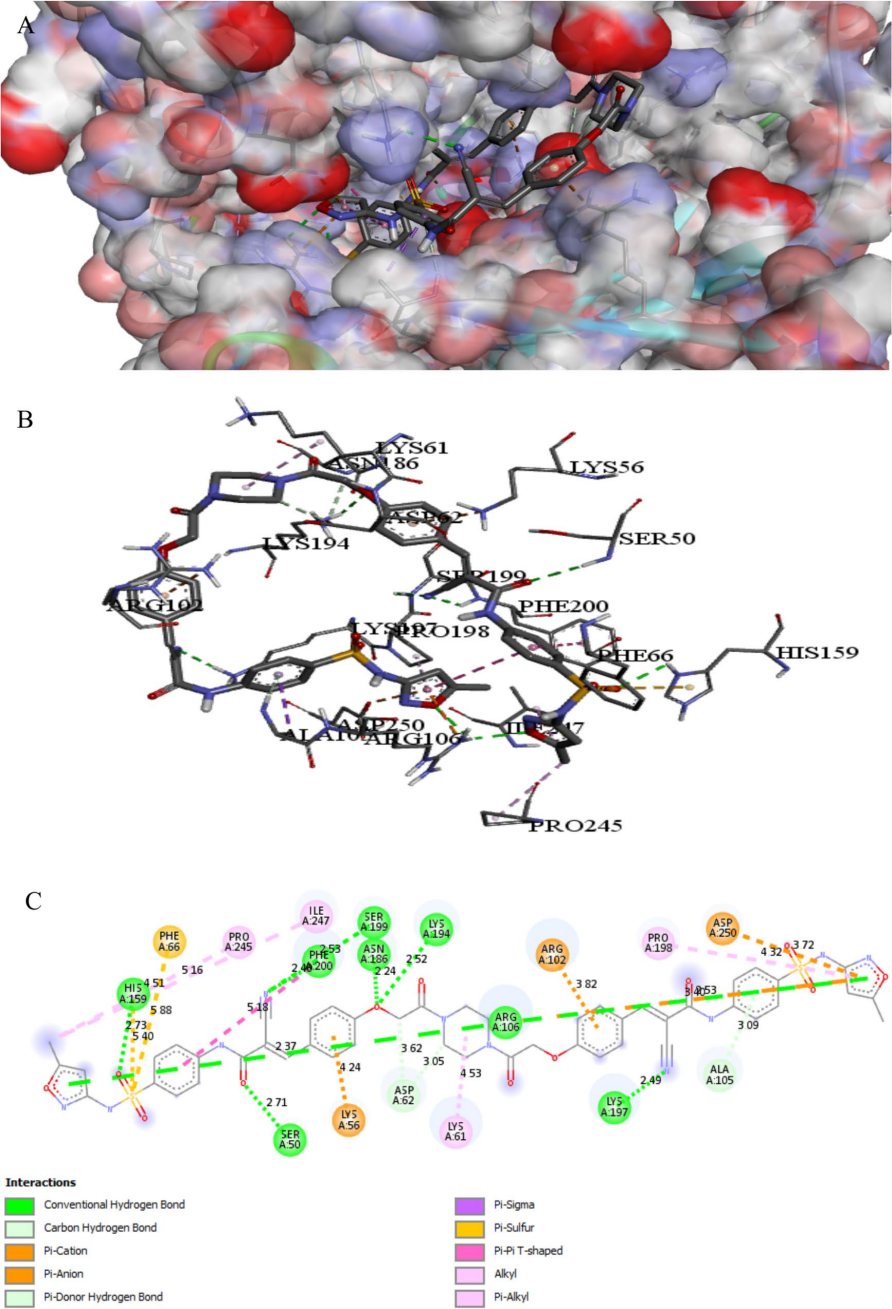
Protein–ligand complex of the tested compound with the active site of Cyclic AMP-AMP-AMP synthase of *Pseudomonas aeruginosa* (UniProt ID: P0DTF7) (**A** and **B**) and 2D interaction of the protein with the compound (**C**)

**Fig. 10:**
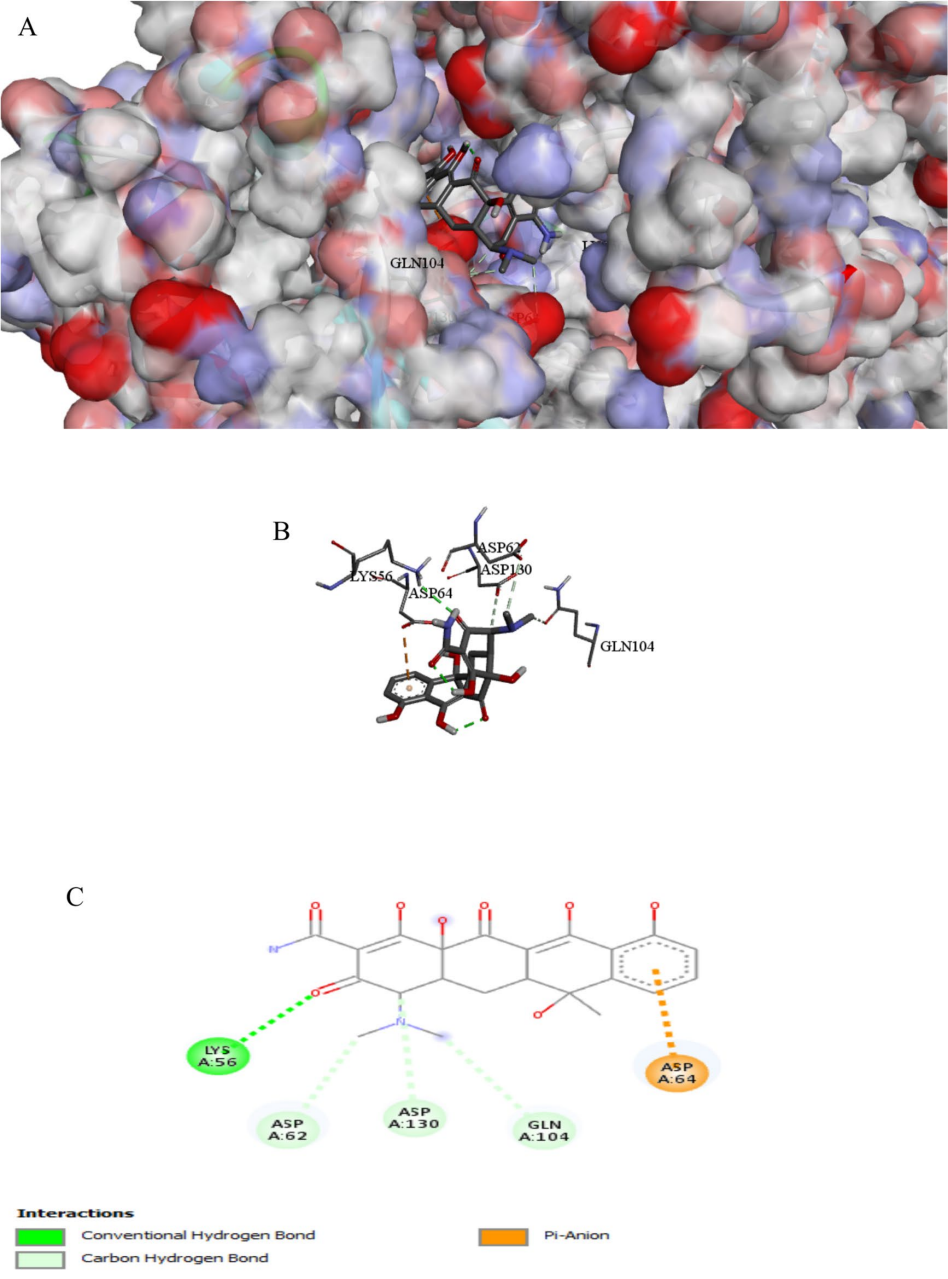
Protein–ligand complex of tetracycline with the active site of Cyclic AMP-AMP-AMP synthase of *Pseudomonas aeruginosa* (UniProt ID: P0DTF7) (**A** and **B**) and 2D interaction of the protein with tetracycline (**C**)

The binding interactions of the tested compound and tetracycline with the active site of the cyclic AMP-AMP-AMP synthase in *Pseudomonas aeruginosa* (UniProt ID: P0DTF7) are illustrated in Figs. 9 and 10. Cyclic AMP regulates various signaling systems, including quorum sensing. Furthermore, cAMP enhances antibiotic resistance pathogenicity and virulence of multidrug-resistant clinical isolates (Harkova et al. 2024). The tested compound showed an interaction with the cyclic AMP-AMP-AMP synthase active site, achieving a binding score of ∆G =  − 10.9 kcal/mol, which is less negative than a binding score of tetracycline (∆G =  − 8.8 kcal/mol). The tested compound strongly bonded to the active site of cyclic AMP through 26 interactions, thirteen of them were conventional H bonds, three Pi-alkyl, one pi-anion, three alkyl, one carbon, four Pi-cation, and one pi-sulfur, while tetracycline showed only five interactions with active sites. The tested compound binds to protein residues of SER107, THR154, ARG102, LYS194, LYS56, ARG255, ARG53, ASP62, HIS159, LYS197, GLN104, SER50, PHE200, LYS197, ARG106, PRO245, LYS194, HIS159, ASP239, PRO198, HIS159, ARG102, ILE247, LYS197, LYS194, and ILE152, while tetracycline binds to amino acids of LYS56, ASP62, GLN104, ASP130, and ASP64 (Table 7). The compound containing the sulfonamide-pyridinyl group forms a hydrogen bond between the oxygen of the sulfonyl group and the side chain of ARG53 (Bishoyi et al. 2022).

**Fig. 11:**
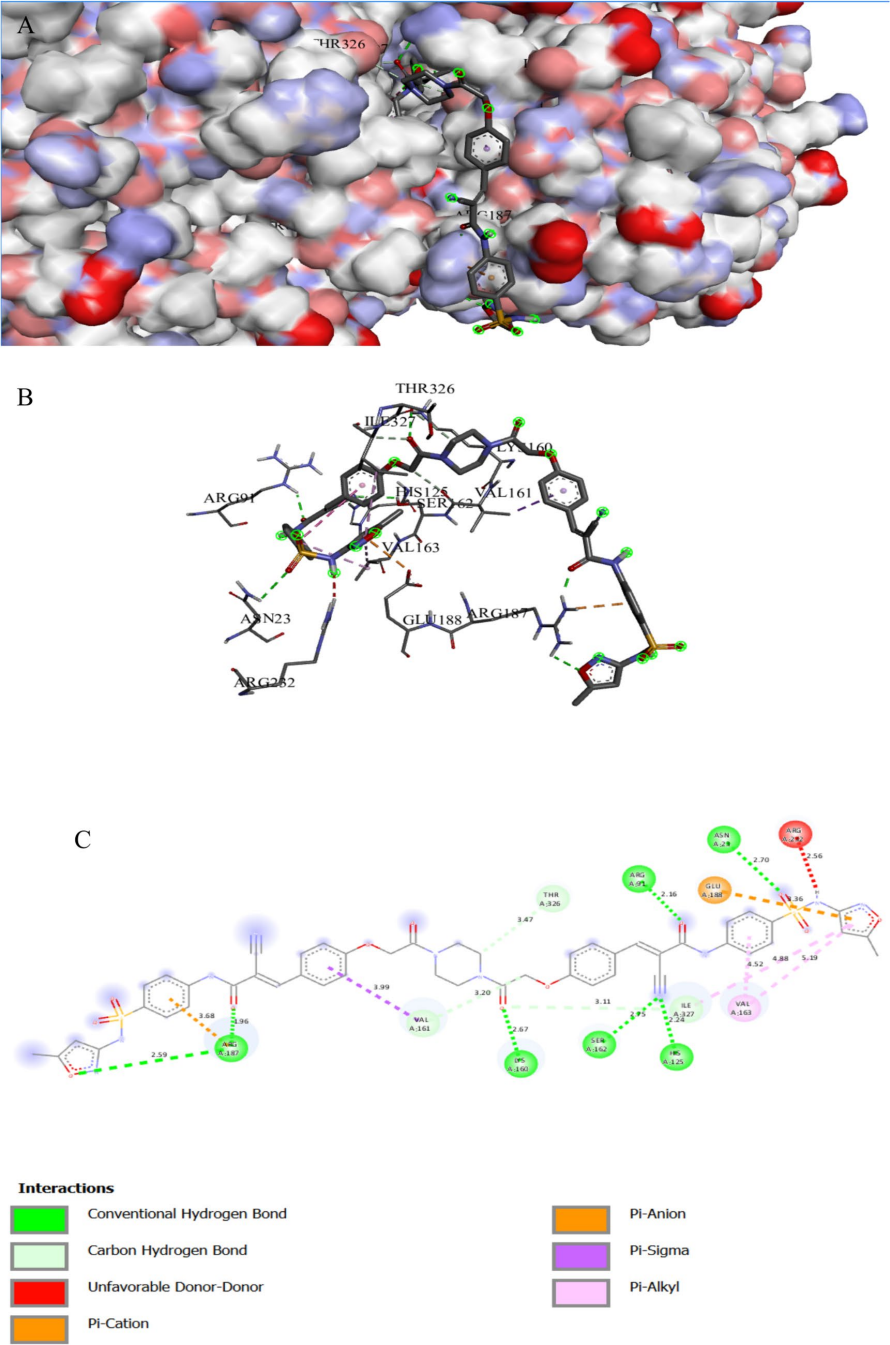
Protein–ligand complex of the tested compound with the active site of UDP-N-acetylglucosamine 1-carboxyvinyltransferase of *Klebsiella pneumoniae* (UniProt ID: A0A1S5RKE3) (**A** and **B**) and 2D interaction of the protein with the compound (**C**)

**Fig. 12:**
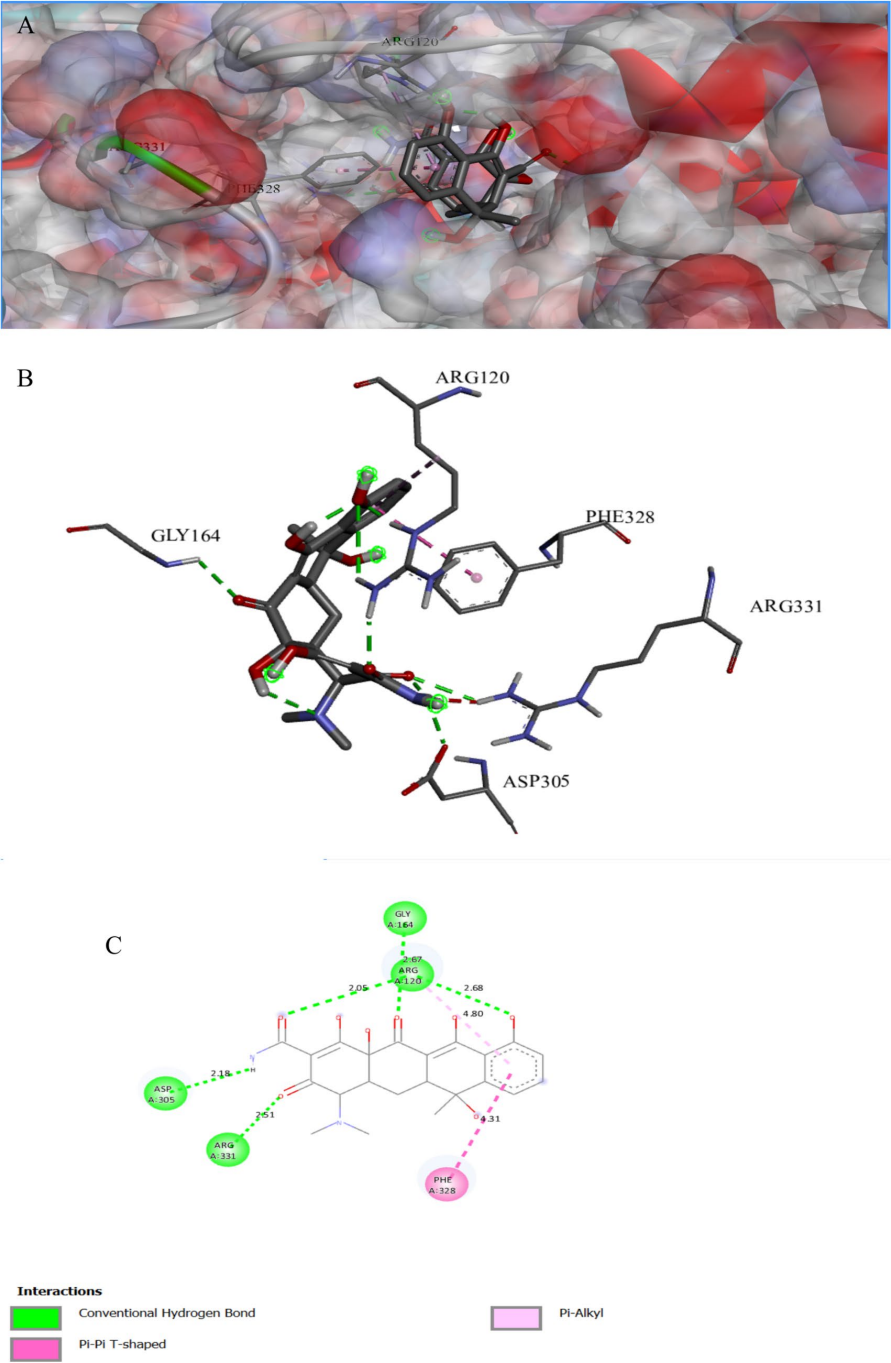
Protein–ligand complex of tetracycline with the active site of UDP-N-acetylglucosamine 1-carboxyvinyltransferase of *Klebsiella pneumoniae* (UniProt ID: A0A1S5RKE3) (**A** and **B**) and 2D interaction of the protein with tetracycline (**C**)

The binding interactions of the tested compound and tetracycline with the active site of the UDP-N-acetylglucosamine 1-carboxyvinyltransferase in *Klebsiella pneumoniae* (UniProt ID: A0A1S5RKE3) are illustrated in Figs. 11 and 12. The UDP-N-acetylglucosamine 1-carboxyvinyltransferase catalyzes the first and second steps of peptidoglycan biosynthesis (Kaur et al. 2021); it also regulates the pathway of cytokinin, which is a hormone that plays an important role in several plant processes such as cell division and differentiation (Jaiswal et al. 2020). The tested compound showed an interaction with the UDP-N-acetylglucosamine 1-carboxyvinyltransferase active site, achieving a binding score of ∆G =  − 9.4 kcal/mol, which is less negative than that of tetracycline (∆G =  − 7.6 kcal/mol). The tested compound bonded tightly to the active site through 16 interactions, seven of them were conventional H bonds, three Pi-alkyl, one pi-anion, three carbon, one Pi-cation, and one pi-sigma, while tetracycline showed only seven interactions with active sites (Table 8). Hydrogen bonding connections are formed between the carbonyl group of an oxygen atom and the hydrogen atoms of LYS160 by a piperazine derivative molecule (Patil et al. 2021).

**Fig. 13:**
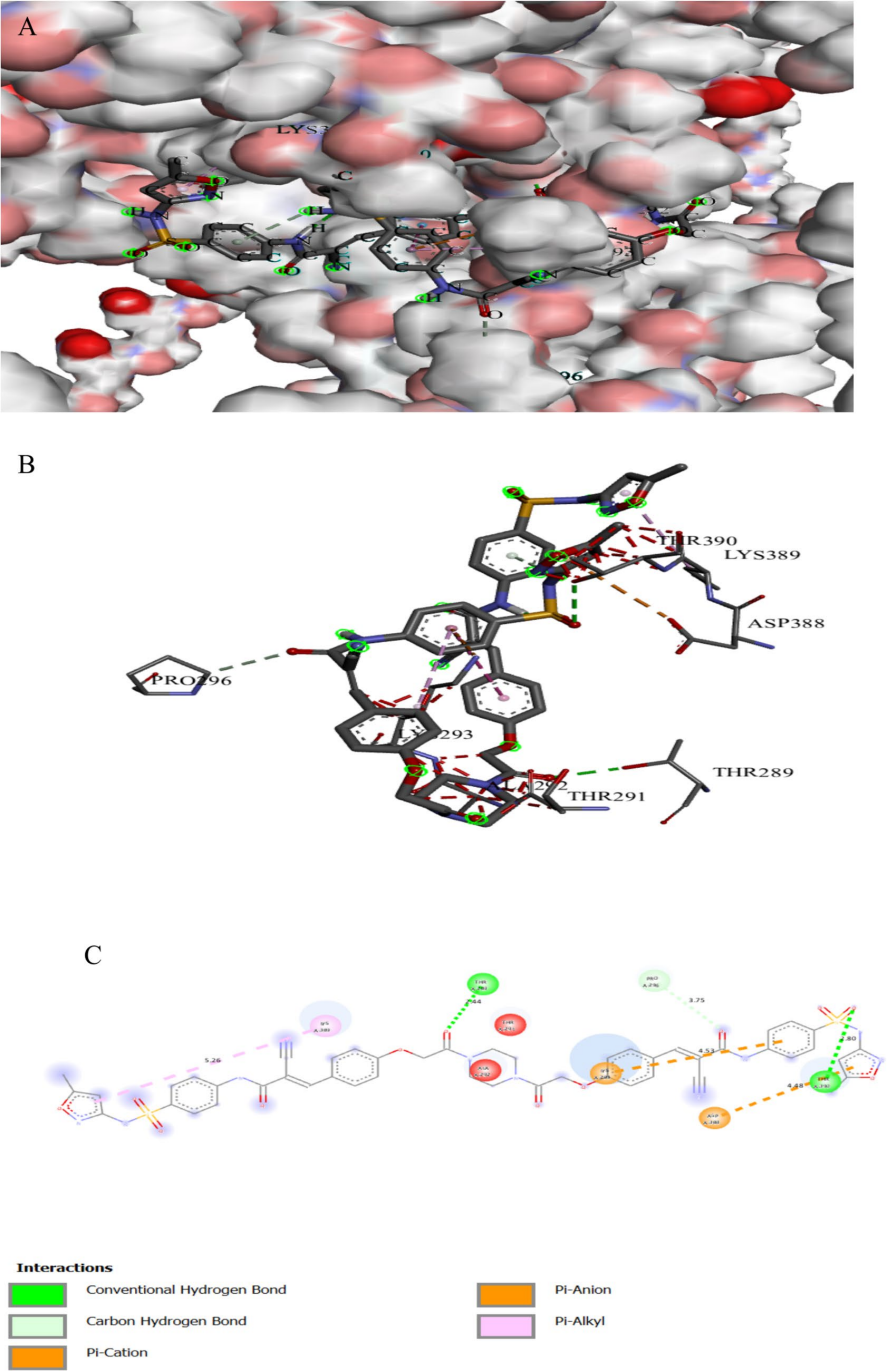
Protein–ligand complex of the tested compound with the active site of clumping factor A in *Staphylococcus aureus* (UniProt ID: Q53653) (**A** and **B**) and 2D interaction of the protein with the compound (**C**)

**Fig. 14:**
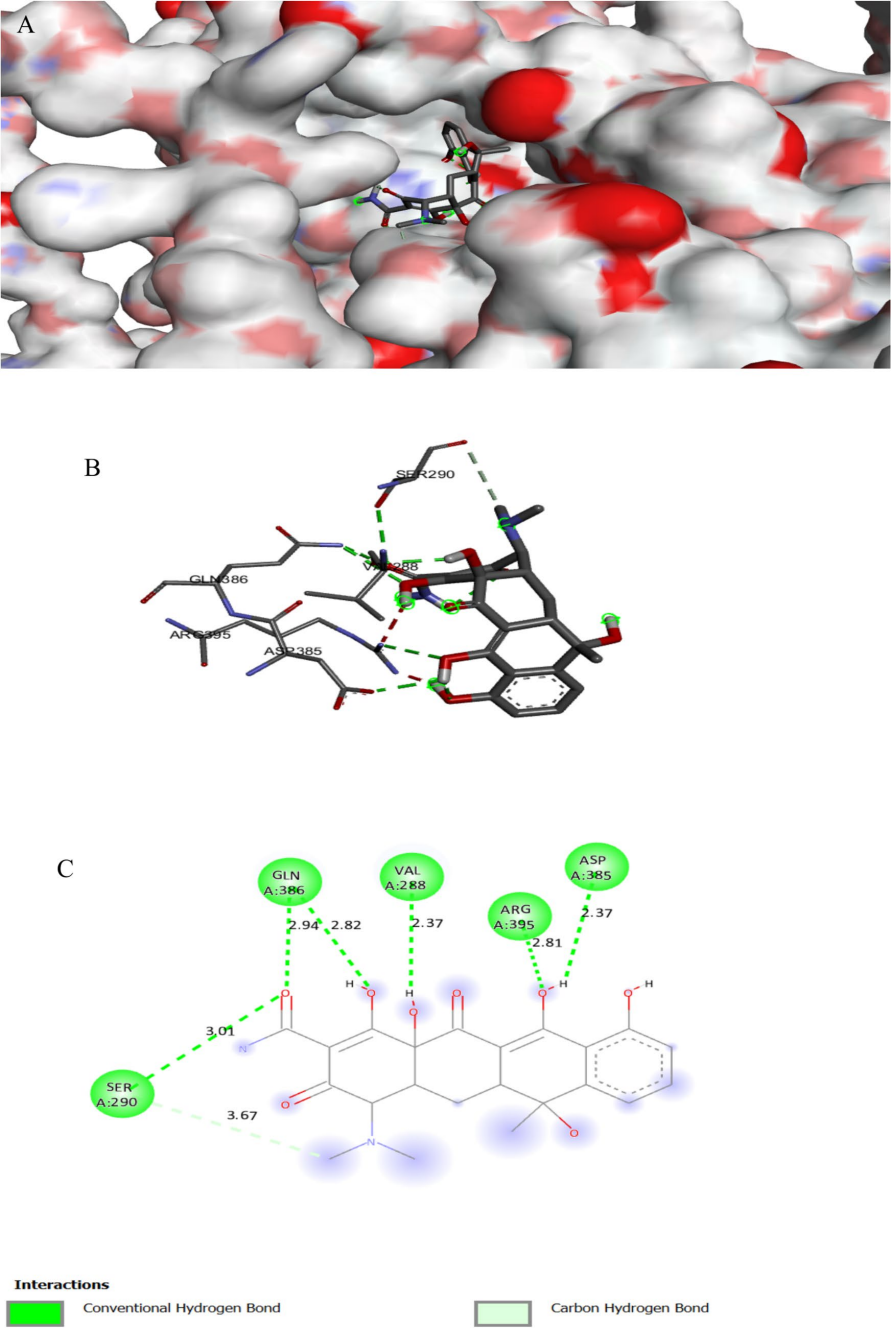
Protein–ligand complex of tetracycline with the active site of clumping factor A in *Staphylococcus aureus* (UniProt ID: Q53653) (**A** and **B**) and 2D interaction of the protein with tetracycline (**C**)

The binding interactions of the tested compound and tetracycline with the active site of the clumping factor A(ClfA) in *Staphylococcus aureus* (UniProt ID: Q53653) are illustrated in Figs. 13 and Fig. 14. ClfA, a surface protein found in *Staphylococcus aureus*, is crucial for ligand binding and adhesion to the endothelium (Hu et al. 2024). *S aureus* has a strong affinity for binding to fibrin and fibrinogen through ClfA. This interaction facilitates the bacteria’s attachment to clots or damaged tissue (Ditkowski et al. 2021). The tested compound showed an interaction with the clumping factor A active site, achieving a binding score of ∆G =  − 9.0 kcal/mol, which is less negative than that of tetracycline (∆G =  − 8.2 kcal/mol). The tested compound bonded tightly to the active site of ClfA through SIX interactions, two of them were conventional H bonds, one Pi-alkyl, and one Pi-cation, while tetracycline showed seven interactions with active sites (Table 9). The tested compound binds to protein residues of THR289, PRO296, ASP388, and LYS 389, while tetracycline binds to amino acids of VAL288, ASP385, ARG395, GLN386, and SER290.

**Table 3:** Specific interaction of the tested compound and fluconazole with secondary metabolism regulator laeA in *Aspergillus niger* (UniProt ID: G3XRG4)

Protein UniProt ID	Compound	Residue involved	Ligand	Interaction type	Distance (A°)
G3XRG4	Tested compound	HIS279	N of cyanide	H-bond	2.17
		ARG331	O of isoxazole	H-bond	2.32
		GLN214	N of cyanide	H-bond	2.43
		SER272	O of sulfonyl group	C-bond	3.37
		CYS207	O of isoxazole	H-bond	3.62
		THR265	π of isoxazole	π-Sigma	3.92
		PRO101	CH_3_ attach of isoxazole	Alkyl	4.05
		TRP213	CH_3_ attach of isoxazole	Alkyl	4.62
		TRP213	π of isoxazole	π–π stacked	4.64
		HIS271	CH_3_ attach of isoxazole	Alkyl	4.64
		CYS207	π of isoxazole	π-Sulfur	4.74
		VAL99	π of benzene	π-Alkyl	4.8
		ARG331	π of isoxazole	π-Alkyl	4.9
		CYS207	CH_3_ attach of isoxazole	Alkyl	5.09
		PRO268	π of isoxazole	π-Alkyl	5.42
		LEU211	π of benzene	π-Alkyl	5.47
		ARG278	N of cyanide	Hydrogen	6.04
	Fluconazole	ASP 166	π of isoxazole	C-bond	3.06
		ARG 111	Carbon	Halogen	3.3
		MET 205	F attach of benzene	π-Cation	3.96
		TYR 100	π of 1,2,4 triazole	π-Alkyl	4.44
		LEU 112	π of 1,2,4 triazole	π-π stacked	4.46
		LEU 102	π of benzene	π-Alkyl	5.1
		LEU 102	π of 1,2,4 triazole	π-Alkyl	5.24

**Table 4:** Specific interaction of the tested compound and fluconazole with Agglutinin-like protein 3 in *Candida albicans* (UniProt ID: O74623)

Protein UniProt ID	Compound	Residue involved	Ligand	Interaction type	Distance (A°)
O74623	Tested compound	TYR243	N of cyanide	H-bond	2.81
		THR313	Carbon	C-bond	2.97
		ASP186	π of benzene	π-Anion	3.45
		TYR38	Carbon	C-bond	3.76
		VAL178	π of benzene	π-Alkyl	4.44
		TRP 312	π of benzene	π-π T-shaped	5.01
		TYR38	π of benzene	π-π T-shaped	5.17
	Fluconazole	ARG 188	F of benzene	H-bond	2.71
		SER 187	H of OH group	H-bond	2.74
		GLY 44	Carbon	C-bond	3.24
		PHE 134	F attach of benzene	Halogen	3.33
		GLY 44	F attach of benzene	Halogen	3.4
		ASN 135	F attach of benzene	Halogen	3.66
		ASP 186	π of pyrazole	π-Anion	3.69
		TYR 38	Carbon	C-bond	3.74
		ARG 188	π of benzene	π-Alkyl	3.99
		PHE 75	π of benzene	π-π T-shaped	5.19

**Table 5:** Specific interaction of the tested compound and tetracycline with DNA gyrase subunit B in *Enterococcus faecalis* (UniProt ID: Q839Z1)

Protein UniProt ID	Compound	Residue involved	Ligand	Interaction type	Distance (A°)
Q839Z1	Tested compound	HIS165	N of cyano gp	H-bond	2.02
		ARG164	O of isoxazole	H-bond	2.32
		SER60	O of sulfonyl	H-bond	2.75
		GLY104	π of isoxazole	π-Hydrogen	2.82
		ARG164	π of isoxazole	π-Cation	2.98
		TYR137	π of alkyl	π-Alkyl	4.03
		GLU52	π of benzene	π-Anion	4.04
		PRO81	π of benzene	π-Alkyl	4.08
		HIS165	π of benzene	π-Cation	4.5
		ARG78	π of benzene	π-Alkyl	4.55
		ARG78	π of benzene	π-Cation	4.56
		LYS105	π of isoxazole	π-Alkyl	4.9
		ILE74	C of CH_3_	Alkyl	5.03
		ARG164	π of benzene	π-Alkyl	5.39
		TYR111	π of benzene	π-π T-shaped	5.72
		TYR111	S of sulfonyl	S of sulfur	5.72
	Tetracycline	HIS278	Oxygen	H-bond	1.98
		HIS40	Oxygen	H-bond	2.47
		TRP43	Oxygen	C-bond	3.64
		ARG192	Oxygen	C-bond	3.77
		ARG192	π of benzene	π-Alkyl	4.7
		TRP43	π of benzene	π-π T-shaped	5.02
		LYS188	π of benzene	π-Alkyl	5.42

**Table 6:** Specific interaction of the tested compound and tetracycline with protein RecA in *Escherichia coli* (UniProt ID: P0A7G6)

Protein UniProt ID	Compound	Residue involved	Ligand	Interaction type	Distance (A°)
P0A7G6	Tested compound	GLU267	N of NH_2_ group	H-bond	2.12
		GLN79	H of NH_2_ group	H-bond	3.04
		GLN262	O of ether	H-bond	3.09
		ARG86	Pi-isoxazole	Pi-cation	3.54
		PHE271	Pi-isoxazole	Pi pi-stacked	3.71
		PHE271	CH_3_ attach of isoxazole	Pi-sigma	4.54
		GLU242	Pi-benzene	Pi-alkyl	4.57
		LAL304	Pi-benzene	Pi-anion	4.8
		LEU278	Pi-isoxazole	Pi-anion	4.9
		LUE264	Pi-benzene	Pi-alkyl	5.32
	Tetracycline	THR75	O of OH	H-bond	2.06
		THR75	H of OH	H-bond	2.58
		THR75	O of carbonyl group	H-bond	2.86
		TYR104	O of OH	H-bond	2.86
		GLY72	O of carbonyl group	C-bond	2.87
		ALA99	H of NH_2_	H-bond	3.0
		SER70	O of OH	H-bond	3.13
		THR74	O of OH	C-bond	3.4
		TYR265	Pi-benzene	Pi pi-stacked	4.15

**Table 7:** Specific interaction of the tested compound and tetracycline with cyclic AMP-AMP-AMP synthase in *Pseudomonas aeruginosa* (UniProt ID: P0DTF7)

Protein UniProt ID	Compound	Residue involved	Ligand	Interaction type	Distance (A°)
P0DTF7	Tested compound	ASN186	O of ether	H-bond	2.24
		ARG106	O of isoxazole	H-bond	2.37
		PHE200	N of Cyano gp	H-bond	2.4
		LYS197	N of Cyano gp	H-bond	2.49
		LYS194	O of ether	H-bond	2.52
		ARG106	O of isoxazole	H-bond	2.53
		SER199	N of Cyano gp	H-bond	2.53
		SER50	O of carbonyl	H-bond	2.71
		HIS159	O of sulfonyl	H-bond	2.73
		ASP62	C of benzene	C-H bond	3.05
		ALA105	π of benzene	π- H-bond	3.09
		ARG106	N of isoxazole	π-Cation	3.4
		ASP62	C of CH_2_	C-H bond	3.62
		ASP250	O of isoxazole	π-Anion	3.72
		ARG102	π of benzene	π-Cation	3.82
		LYS56	π of benzene	π-Cation	4.24
		PRO198	π of isoxazole	π-Alkyl	4.32
		PRO245	CH3 group	Alkyl	4.51
		LYS61	π of piperazine	Alkyl	4.53
		ILE247	π of CH_3_	Alkyl	5.16
		PHE200	π of benzene	π-π T-shaped	5.18
		HIS159	π of sulfonyl	S of sulfur	5.4
		PHE66	π of sulfonyl	S of sulfur	5.88
	Tetracycline	LYS56	O of carbonyl group	H-bond	2.57
		ASP62	CH_3_ group	C-bond	3.22
		GLN104	CH_3_ group	C-bond	3.5
		ASP130	CH_2_ group	C-bond	3.59
		ASP64	π of benzene	π-Anion	3.89

**Table 8:** Specific interaction of the tested compound and tetracycline with UDP-N-acetylglucosamine 1-carboxyvinyltransferase of *Klebsiella pneumoniae* (UniProt ID: A0A1S5RKE3)

Protein UniProt ID	Compound	Residue involved	Ligand	Interaction type	Distance (A°)
A0A1S5RKE3	Tested compound	ARG 187	O of carbonyl group	H-bond	1.96
		ARG 91	O of carbonyl group	H-bond	2.16
		HIS 125	N of cyanide group	H-bond	2.24
		ARG 187	O of isoxazole	H-bond	2.59
		LYS 160	O of carbonyl group	H-bond	2.67
		ASN 23	O of carbonyl group	H-bond	2.7
		SER 162	N of cyanide group	H-bond	2.75
		ILE 327	O of carbonyl group	C-bond	3.11
		VAL 161	CH_2_ group	C-bond	3.2
		GLU188	π of isoxazole	π-Anion	3.36
		THR 326	C of piperazine	C-bond	3.47
		ARG 187	π of benzene	π -Cation	3.68
		VAL 161	π of benzene	π-Sigma	3.9
		VAL 163	π of benzene	π-Alkyl	4.52
		ILE 327	π of isoxazole	π-Alkyl	4.52
		VAL 163	π of isoxazole	π-Alkyl	5.19
	Tetracycline	ARG120	O of carbonyl group	H-bond	2.05
		ASP 305	H of NH group	H-bond	2.18
		ARG 331	O of carbonyl group	H-bond	2.51
		GLY 164	O of carbonyl group	H-bond	2.67
		ARG120	O attach of benzene	H-bond	2.68
		PHE 328	π of benzene	π-π T-shaped	4.31
		ARG120	π of benzene	π-Alkyl	4.8

**Table 9:** Specific interaction of the tested compound and tetracycline with clumping factor A in *Staphylococcus aureus* (UniProt ID: Q53653)

Protein UniProt ID	Compound	Residue involved	Ligand	Interaction type	Distance (A°)
Q53653	Tested compound	THR289	C of carbonyl	H-bond	2.44
		THR390	O of sulfonyl group	H-bond	2.80
		PRO296	C of carbonyl	C-bond	3.75
		ASP388	π of isoxazole	Pi-cation	4.48
		LYS293	Pi-benzene	Pi-cation	4.53
		LYS 389	π of isoxazole	Pi-alkyl	5.26
	Tetracycline	VAL288	H of OH	H-bond	2.27
		ASP385	H of OH	H-bond	2.37
		ARG395	O of carbonyl	H-bond	2.81
		GLN386	O of carbonyl	H-bond	2.82
		GLN386	O of carbonyl	H-bond	2.94
		SER290	O of carbonyl	H-bond	3.01
		SER290	CH_3_ of N	H-bond	3.67

## Conclusion

Multiple factors play a role in the pathogenesis of rheumatoid arthritis (RA), with infections being a significant concern for patients, as they substantially contribute to mortality risk. Consequently, there is a pressing need for effective strategies to develop innovative preventive measures and therapeutic interventions to modify the course of RA. The synthesized cyanoacrylamide-based piperazine containing sulphamethoxazole moiety demonstrated potent antibacterial activity and low antifungal activity against bacterial and fungal pathogens. *S. aureus* exhibited the highest inhibition, with a zone diameter of 16.0 ± 1.0 mm at a concentration of 0.8 mg/ml, while the MIC for all bacterial species ranged from 5 to 40 mg/ml. Fungal species, however, showed the highest resistance to the compound. Additionally, molecular docking studies against seven key microbial proteins revealed a high binding affinity, suggesting a strong interaction with crucial molecular targets. The compound achieved the highest binding score of ∆G =  − 10.9 kcal/mol at the cyclic AMP synthase active site (UniProt ID: P0DTF7) of *P. aeruginosa*, forming 26 interactions. This dual approach of experimental validation and computational analysis underscores the compound’s therapeutic potential. It provides valuable insights into its mechanism of action, paving the way for its development as a novel antimicrobial agent. Given the promising biological properties of this class of compounds, further research focusing on structural modifications and substitutions within the base molecule is recommended to optimize its antimicrobial efficacy, particularly against bacterial and fungal pathogens implicated in rheumatoid arthritis.

## Supplementary Information

Below is the link to the electronic supplementary material.

Supplementary file1 (DOCX 581 KB)

## Data Availability

All source data for this work (or generated in this study) are available upon reasonable request.
