# Supplementary material for: Synthesis and antimicrobial evaluation of a new hybrid bis-cyanoacrylamide-based-piperazine containing sulphamethoxazole moiety against rheumatoid arthritis-associated pathogens
Source: Naunyn Schmiedebergs Arch Pharmacol. 2025 Jan 20;398(7):8587–617. doi: 10.1007/s00210-024-03780-7 (PMC12263765; doi:10.1007/s00210-024-03780-7)
Supplement: Supplementary file 1 — Supplementary file1 (DOCX 581 KB) [file 210_2024_3780_MOESM1_ESM.docx]

**Synthesis and Antimicrobial Evaluation of a New Hybrid Bis-Cyanoacrylamide-Based-Piperazine Containing Sulphamethoxazole Moiety Against Rheumatoid Arthritis-Associated Pathogens**

Mona M. Soliman,^1^ Ahmed M. Sayed,^1^ Mahmoud Ibrahim,^1^ Mohamed A Dawoud,^1^ Shahd Hisham Mohamed Ali,^1^ Menna Tallah S. Nady,^2^ Nada A. Hassan,^3^ Wessam Saad,^2^ Ahmed H. M. Elwahy,^4^ Ismail A. Abdelhamid^4^

^1^Department of Botany and Microbiology, Faculty of Science, Cairo University, Giza, 12613, Egypt

^2^Department of Biotechnology, Faculty of Agriculture, Cairo university, Giza, 12613, Egypt

^3^Department of Zoology, Faculty of Science, Cairo university, Giza, 12613, Egypt

^4^Department of Chemistry, Faculty of Science, Cairo University, Giza 12613, Egypt.

[aelwahy@hotmail.com](mailto:aelwahy@hotmail.com), [ismail_shafy@yahoo.com](mailto:ismail_shafy@yahoo.com); [ismail_shafy@cu.edu.eg](mailto:ismail_shafy@cu.edu.eg)

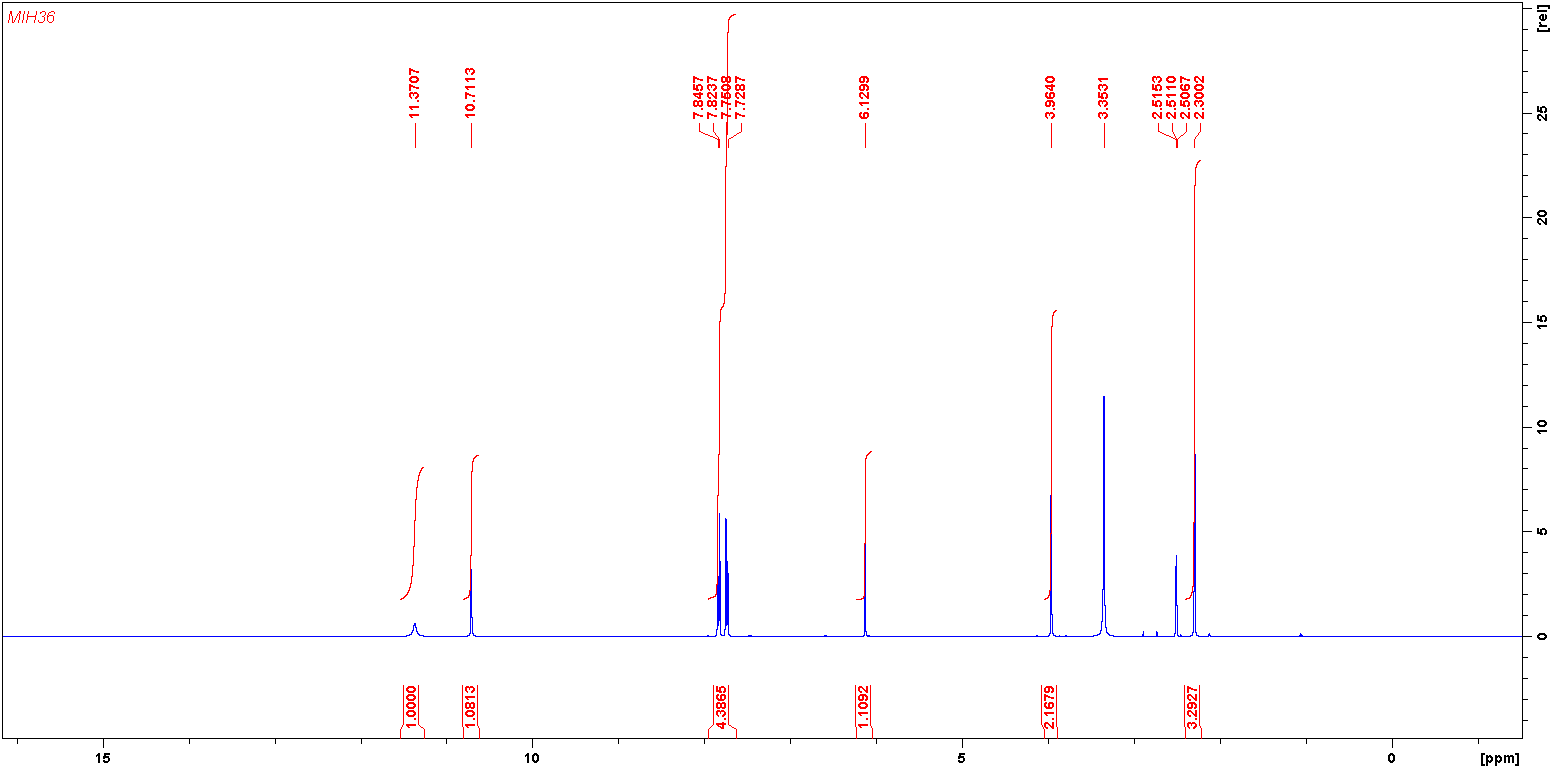


^1^H NMR spectrum of compound **3**

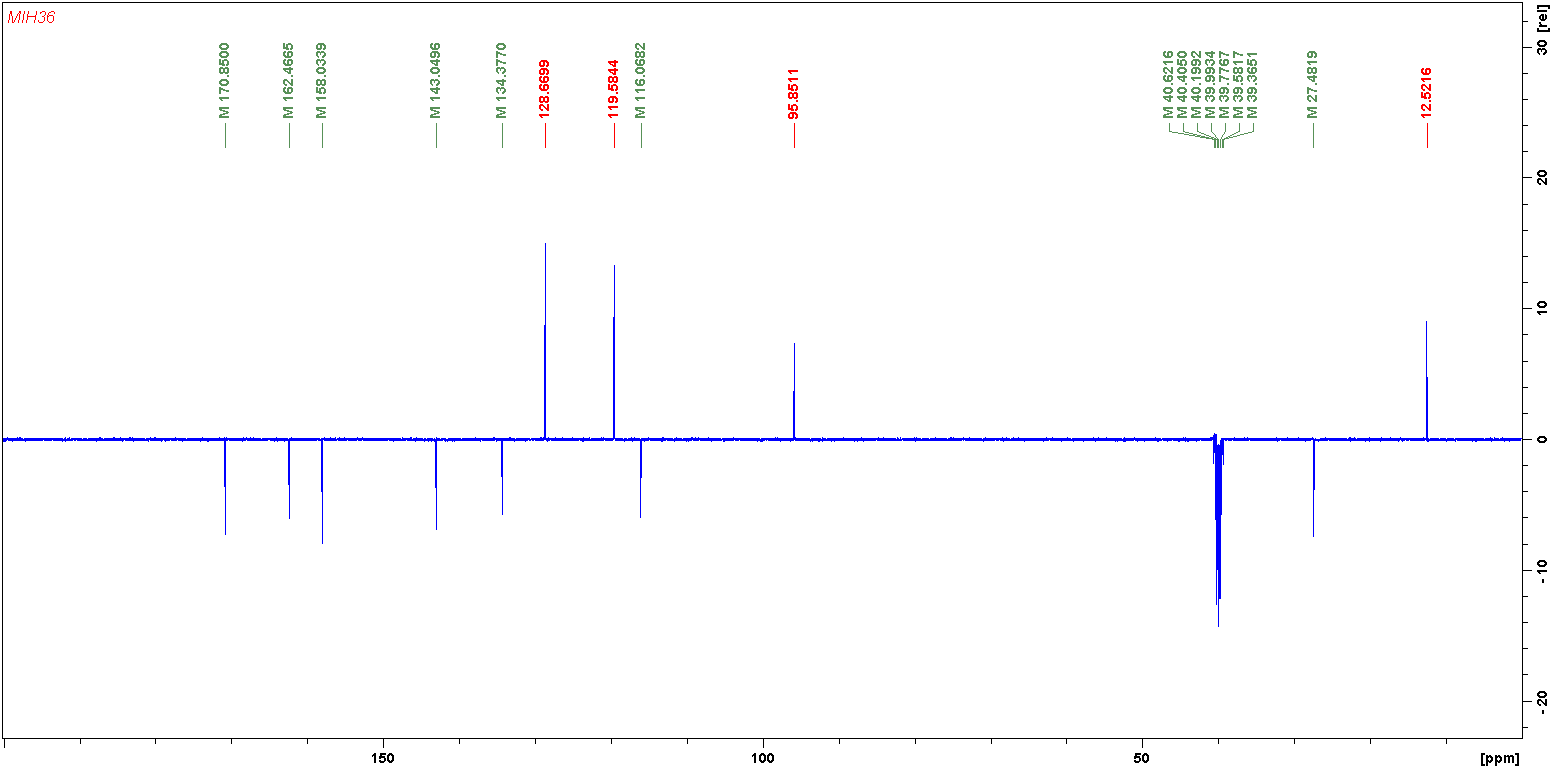


^13^C NMR spectrum of compound **3**


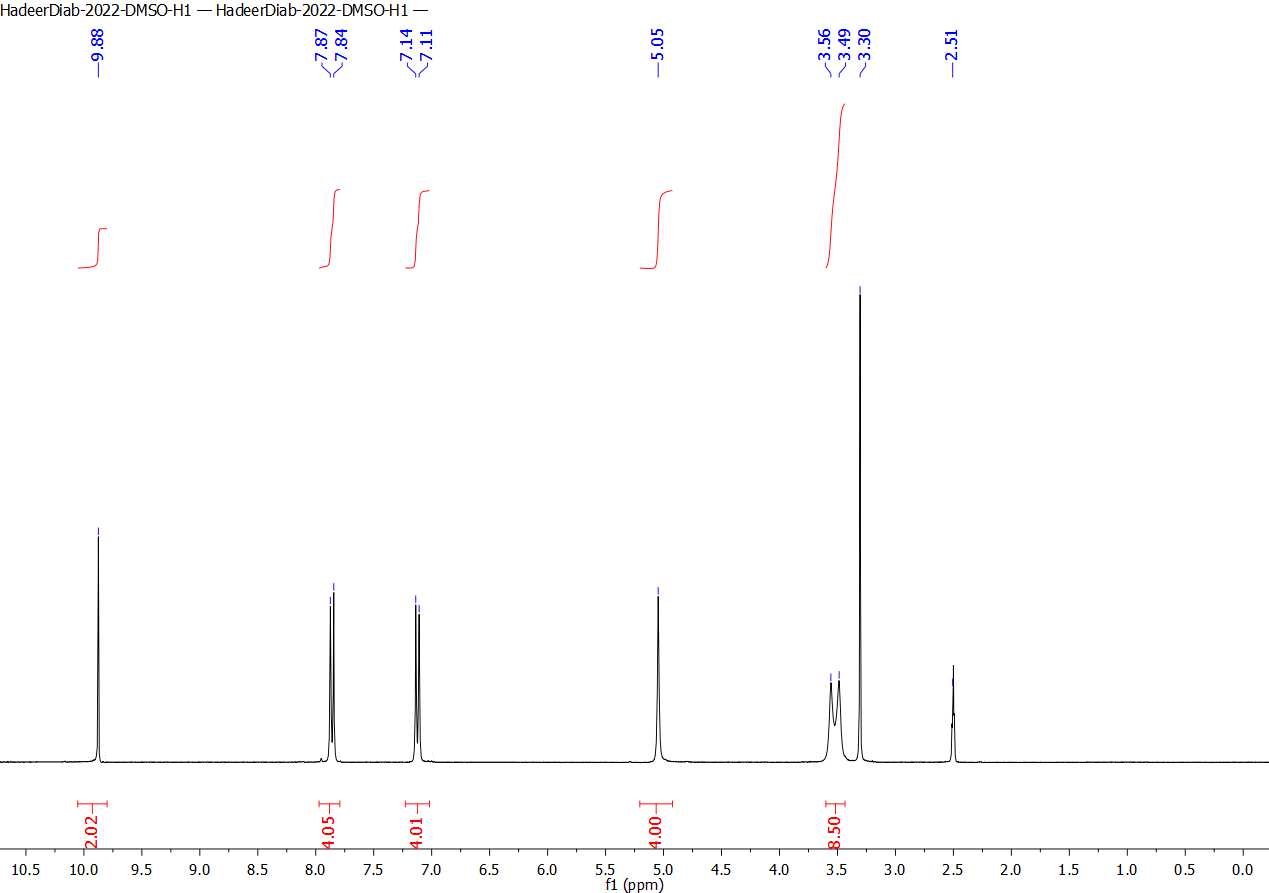

^1^H NMR spectrum of compound **8**


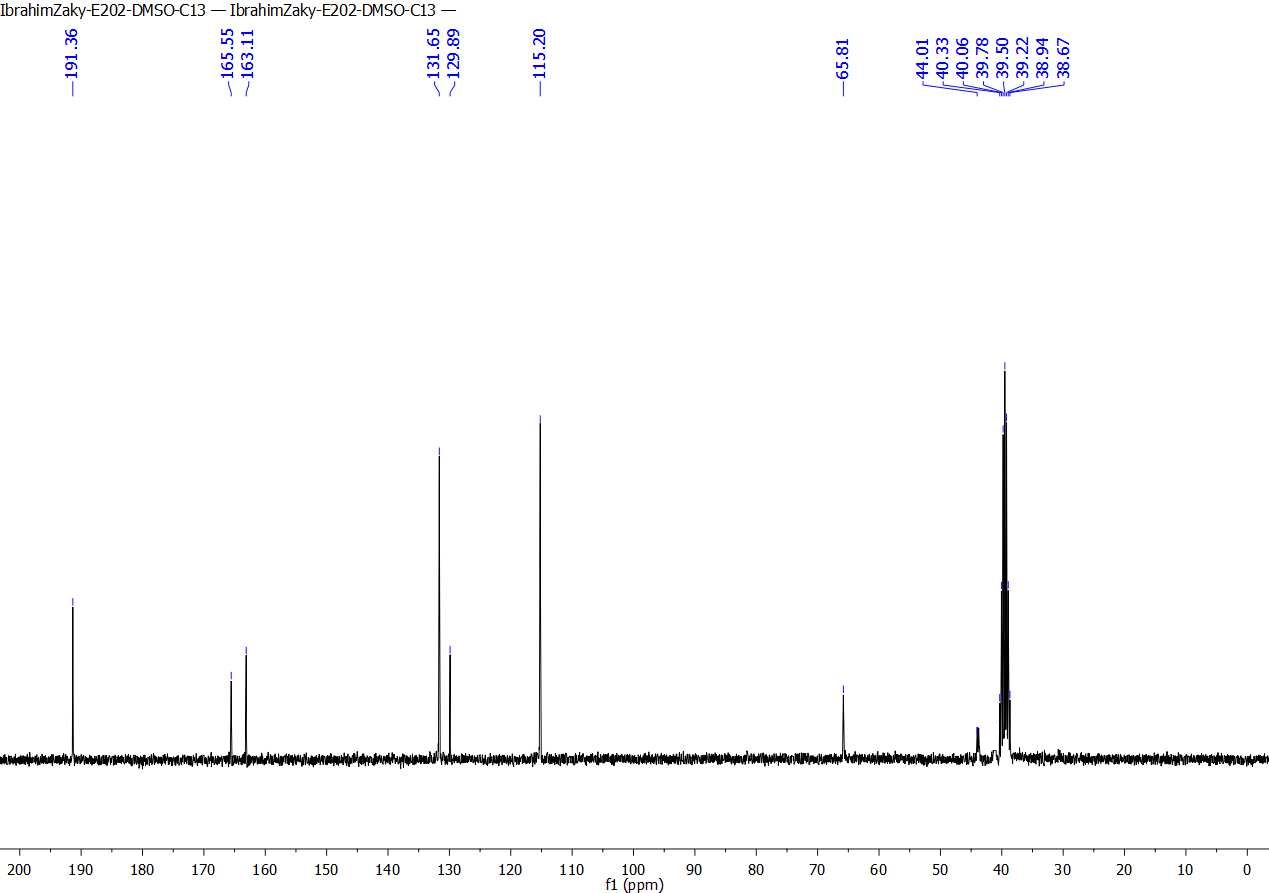

^13^C NMR spectrum of compound **8**


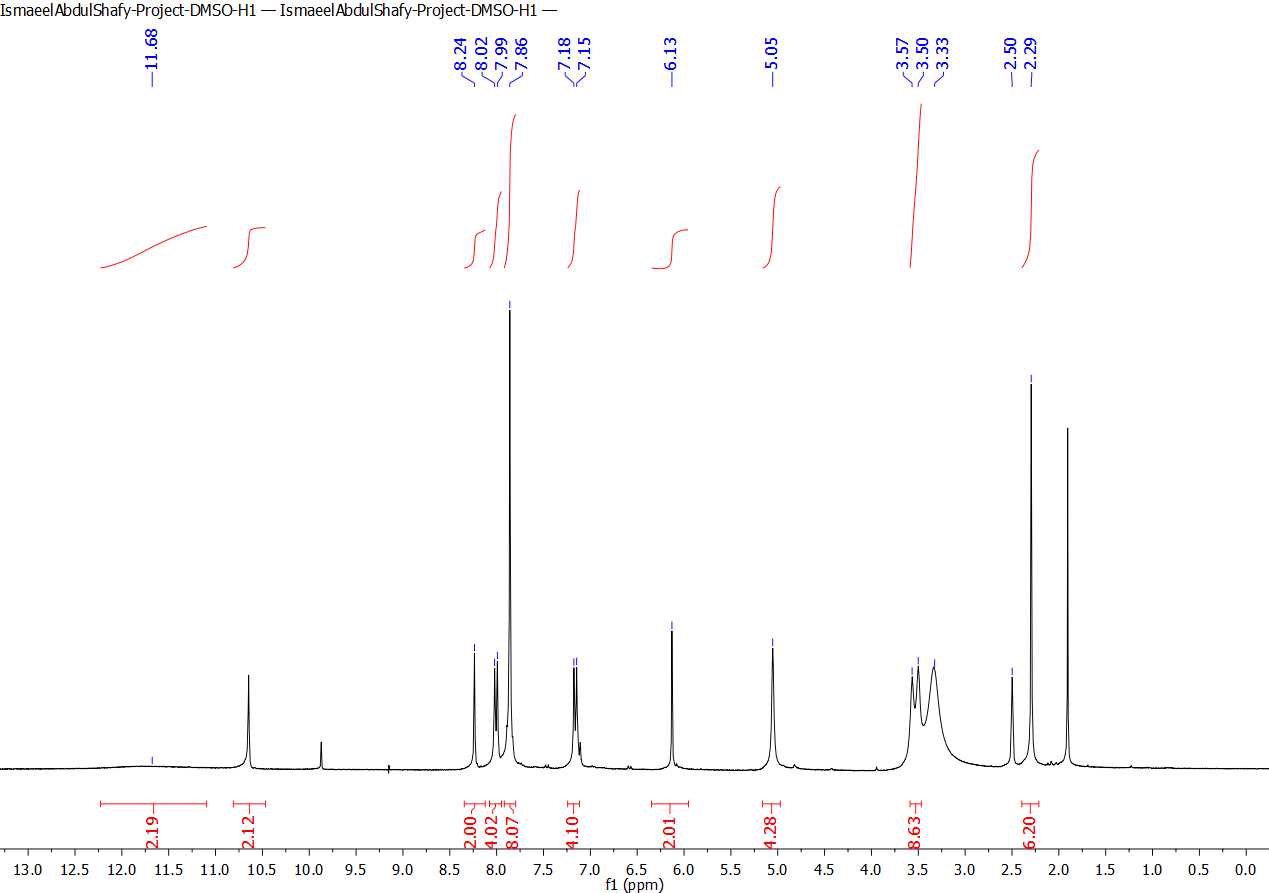

**^1^H NMR Spectrum of compound 9**


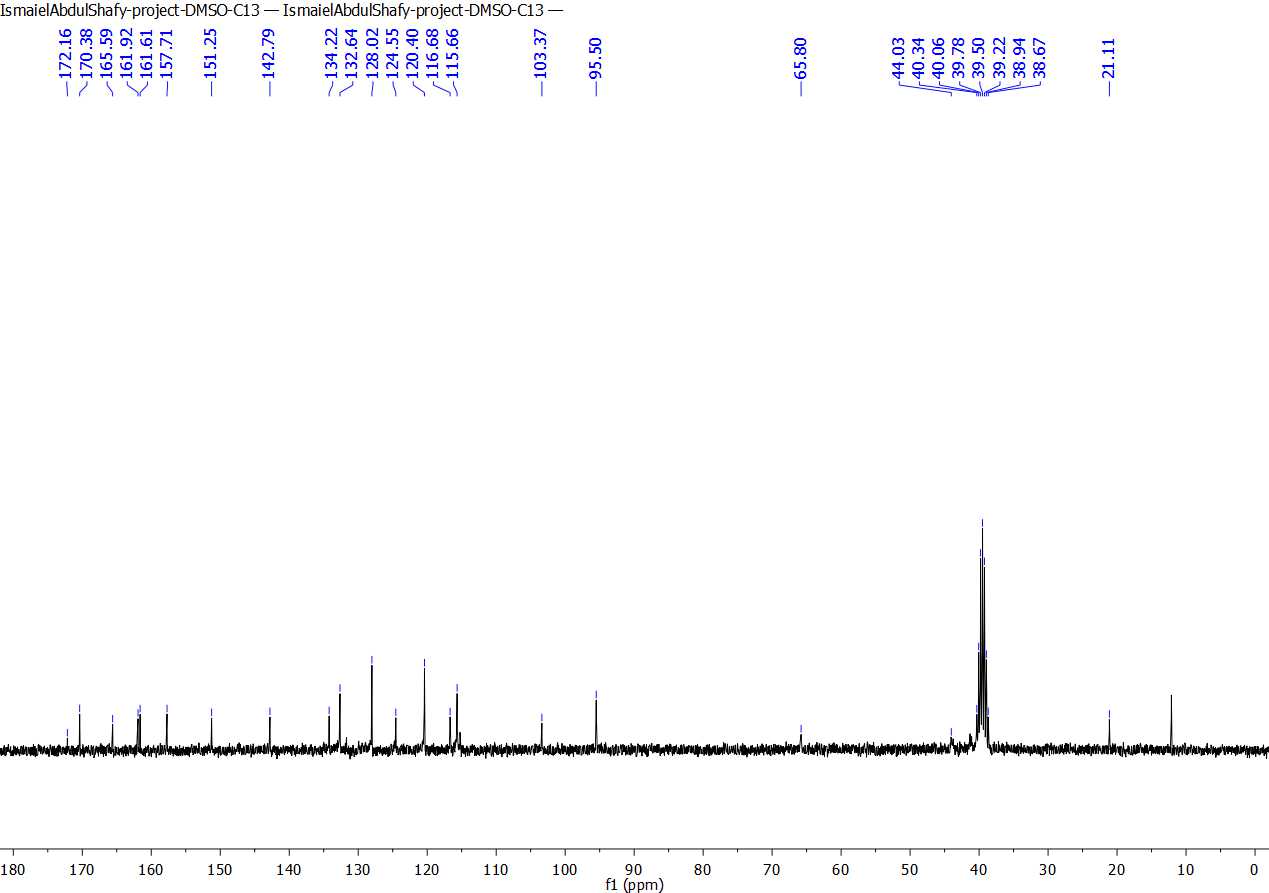

**^13^C NMR Spectrum of compound 9**


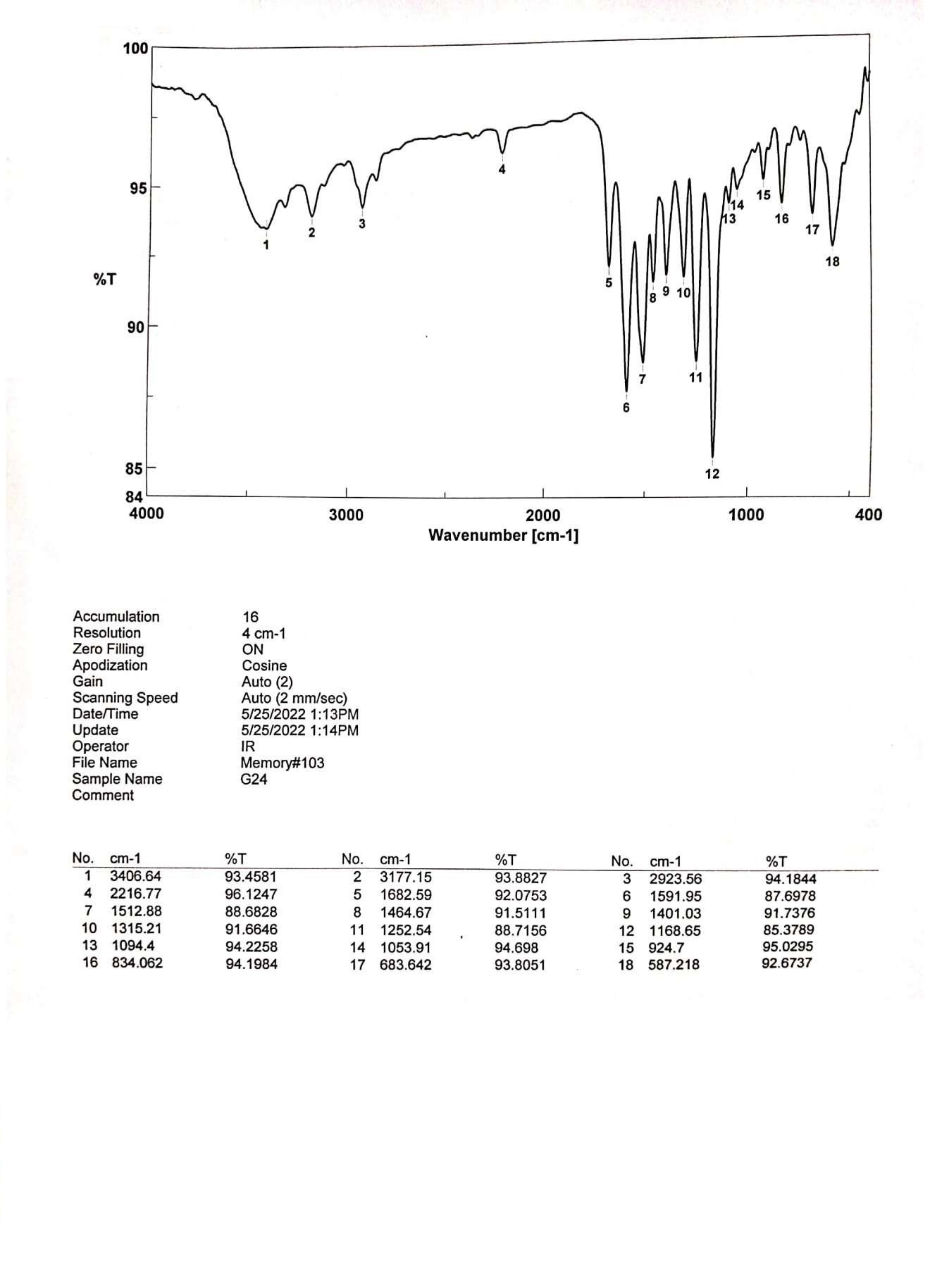


**IR Spectrum of compound 9**
